# Promoter-Specific Expression and Genomic Structure of IgLON Family Genes in Mouse

**DOI:** 10.3389/fnins.2017.00038

**Published:** 2017-02-02

**Authors:** Taavi Vanaveski, Katyayani Singh, Jane Narvik, Kattri-Liis Eskla, Tanel Visnapuu, Indrek Heinla, Mohan Jayaram, Jürgen Innos, Kersti Lilleväli, Mari-Anne Philips, Eero Vasar

**Affiliations:** ^1^Department of Physiology, Institute of Biomedicine and Translational Medicine, University of TartuTartu, Estonia; ^2^Division of Pharmacology and Pharmacotherapy, Faculty of Pharmacy, University of HelsinkiHelsinki, Finland

**Keywords:** IgLON, OPCML, NTM, LSAMP, NEGR1, KILON, IgLON5, alternative promoters

## Abstract

IgLON family is composed of five genes: Lsamp, Ntm, Opcml, Negr1, and Iglon5; encoding for five highly homologous neural adhesion proteins that regulate neurite outgrowth and synapse formation. In the current study we performed *in silico* analysis revealing that Ntm and Opcml display similar genomic structure as previously reported for Lsamp, characterized by two alternative promotors 1a and 1b. Negr1 and Iglon5 transcripts have uniform 5′ region, suggesting single promoter. Iglon5, the recently characterized family member, shares high level of conservation and structural qualities characteristic to IgLON family such as N-terminal signal peptide, three Ig domains, and GPI anchor binding site. By using custom 5′-isoform-specific TaqMan gene-expression assay, we demonstrated heterogeneous expression of IgLON transcripts in different areas of mouse brain and several-fold lower expression in selected tissues outside central nervous system. As an example, the expression of IgLON transcripts in urogenital and reproductive system is in line with repeated reports of urogenital tumors accompanied by mutations in IgLON genes. Considering the high levels of intra-family homology shared by IgLONs, we investigated potential compensatory effects at the level of IgLON isoforms in the brains of mice deficient of one or two family members. We found that the lack of IgLONs is not compensated by a systematic quantitative increase of the other family members. On the contrary, the expression of Ntm 1a transcript and NEGR1 protein was significantly reduced in the frontal cortex of Lsamp-deficient mice suggesting that the expression patterns within IgLON family are balanced coherently. The actions of individual IgLONs, however, can be antagonistic as demonstrated by differential expression of Syp in deletion mutants of IgLONs. In conclusion, we show that the genomic twin-promoter structure has impact on both anatomical distribution and intra-family interactions of IgLON family members. Remarkable variety in the activity levels of 1a and 1b promoters both in the brain and in other tissues, suggests complex functional regulation of IgLONs by alternative signal peptides driven by 1a and 1b promoters.

## Introduction

IgLON family is composed of five neural adhesion proteins: OPCML (OBCAM; IgLON1; Schofield et al., 1989), NTM (IgLON2; Struyk et al., 1995), LSAMP (IgLON3; Horton and Levitt, 1988), NEGR1 (KILON; IgLON4; Funatsu et al., 1999), and IgLON5 (Grimwood et al., 2004; Sabater et al., 2014) (names are based on HGNC nomenclature; http://www.genenames.org/). IgLON family members are characterized by three Ig domains and glycosylphosphatidylinositol (GPI) anchor (Sellar et al., 2003; Pimenta and Levitt, 2004). IgLONs form homo- and heterophilic dimers in *cis* within family and act in *trans* (between cells) on the plane of the membrane as part of a larger signaling complex (Reed et al., 2004; McNamee et al., 2011). All IgLON family members are expressed both in neurons and oligodendrocytes except for NTM, which has been found to express specifically in neurons (Sharma et al., 2015). OPCML, NTM, LSAMP, and NEGR1 regulate neurite outgrowth and participate in synapse formation (Struyk et al., 1995; Mann et al., 1998; Gil et al., 2002; Hashimoto et al., 2009; Sugimoto et al., 2010; Akeel et al., 2011). Surprisingly, accumulating data confirms that IgLON neural adhesion molecules can function as tumor-suppressor genes in a number of non-neural organs and tissue types (Sellar et al., 2003; Ntougkos et al., 2005; Reed et al., 2007; Cui et al., 2008; Barøy et al., 2014; Kim et al., 2014). The early onset of expression during embryogenesis and continuous requirement in adulthood suggests that IgLONs have important roles throughout the life cycle and are implicated in disease states (Schwarz et al., 2009). For example, polymorphisms in *Negr1* gene have been associated with white matter integrity (Dennis et al., 2014), depression (Hyde et al., 2016), and obesity (Melén et al., 2010; Hotta et al., 2011; Elks et al., 2012; Poveda et al., 2014) in several genome wide association studies. LSAMP is implicated in the regulation of emotional and social behavior in mouse models (Innos et al., 2011, 2012, 2013b; Philips et al., 2015) and has been associated with psychiatric disorders such as major depressive disorder and schizophrenia in humans (Behan et al., 2009; Koido et al., 2012, 2014; Innos et al., 2013a). A number of publications emphasize the role of OPCML as tumor-suppressor in different tumor types (Cui et al., 2008; McKie et al., 2012). Polymorphisms in *NTM* gene, the closest kindred to OPCML, have been associated with intelligence (Pan et al., 2011) and shown to participate in axon fasciculation (Chen et al., 2001; Yu et al., 2012). *Iglon5*, a newly discovered member of the IgLON family, has a role in encephalopathy with prominent sleep dysfunction, chronic neurodegeneration, and cell-surface autoimmunity (Sabater et al., 2014; Leypoldt et al., 2015).

We have previously demonstrated differential promoter activity of two alternative promoters in *Lsamp* gene (Philips et al., 2015). The expression pattern of two promoters diverges from the developmental initiation to adulthood and is likely related to the fine tuning of neural pathways during development. The expression of 1a isoform of *Lsamp* gene is dominant in “classic” limbic structures, particularly in the hippocampus and amygdaloid area. 1b isoform is prevalent in the sensory nuclei of thalamus and cortical sensory areas (Philips et al., 2015). These findings underline the importance of studying 1a and 1b promoter activities independently in the IgLON family. The sequences of 1a and 1b transcripts of *Lsamp* gene in human, mouse, and rat were initially described by Pimenta and Levitt (2004). However, no valid analysis of the structure and possible alternative promoters exists for other genes of the IgLON family.

Bioinformatic analysis of first exon structure of IgLON genes for mouse, rat, and human was carried out to ascertain the sequences and the number of active promoters for each gene. We thereafter designed a customized 5′-isoform-specific quantitative real-time PCR (qPCR) assay to distinguish between alternative promoter products of *Opcml, Ntm*, and *Lsamp* in mice. Our study provides the first comprehensive characterization of the IgLON family transcription pattern in the mouse brain. Considering that the tumor-suppressor role of IgLON proteins has repeatedly been affirmed in a variety of organs, we also evaluated the expression of IgLON transcripts in 12 non-neural tissues. Although IgLONs have been reported to have essential function in development of neural cirquits, no gross anatomy changes have been reported in the brains of mice deficient for IgLON proteins (Lee et al., 2012; Philips et al., 2015; Mazitov et al., 2017). Considering the high levels of intra-family homology shared by IgLON adhesion molecules, potential compensatory effects within IgLON gene family were also studied: we estimated promoter activities in the frontal cortex and hippocampi of mice with deletion mutation in *Lsamp* (*Lsamp*^−/−^), *Ntm* (*Ntm*^−/−^), or in both (*Lsamp*^−/−^/*Ntm*^−/−^) genes. Finally, the expression of *Synaptophysin*, a marker of synaptogenesis, was studied in the same mutant mice to gain insight to the intra-family interactions in functional systems.

## Materials and methods

### *In silico* analysis of alternative first exons and protein domains

**Figure 1:**
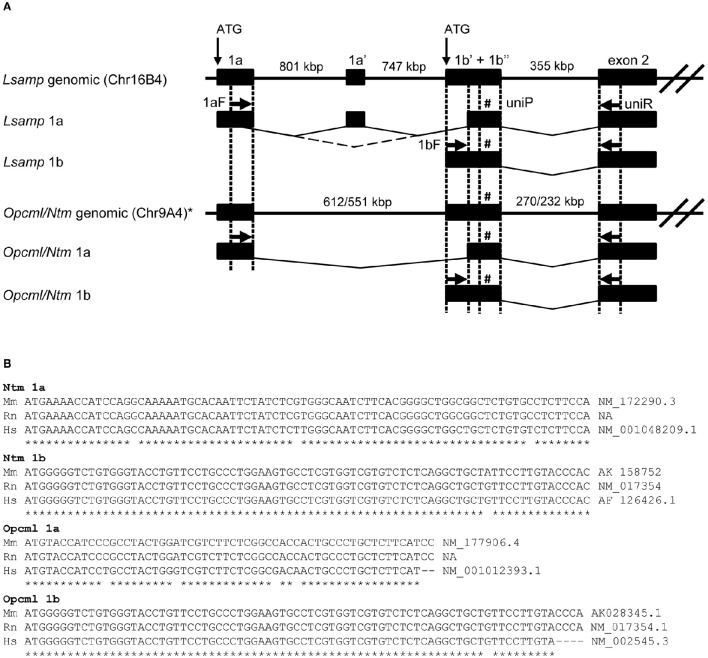
**Twin promoter structure of ***Opcml***, ***Ntm***, and ***Lsamp*** genes and alignment of alternative 5′ fragments of the coding region. (A)** Genomic structure and assembly of the 5′ part of 1a and 1b promoter derived transcripts. The core of the schema has been adapted from Pimenta and Levitt (2004). The 5′ part of 1b transcript is assembled by adding up 1b' + 1b” + exon 2, followed by the downstream exons in both rodent and human. The 5′ part of 1a transcript is typically assembled by adding up 1a + 1b” + exon 2, followed by the downstream exons. Exception of this is *Lsamp* 1a transcript in rodents where one extra exon (1a') is included: 1a +1a' +1b” + exon 2. Horizontal arrows denoted as 1aF and 1bF indicate transcript specific forward primers on 1a and 1b first exon respectively. Horizontal arrow denoted as uniR indicates universal reverse primer on exon 2 and # denoted as uniP indicates custom probe on 1b”. ^*^Ntm and *Opcml* are located in the same chromosome locus. **(B)** Alignment of unique 1a and 1b transcript specific 5′ sequences of *Ntm* and *Opcml* in rodent and human with corresponding Refseq or Non-Refseq accession numbers. NA refers to “accession number not available,” indicating rat 1a sequences that were missing from the NCBI/Ensembl.org database at the time of the analysis and were therefore amplified and analyzed for the current study. More information about accession numbers can be found in Supplementary Tables S1–S3.

Bioinformatic analysis of alternative first exons and respective upstream regions (5′UTR) of rodent and human *Opcml* and *Ntm* transcripts was performed by detecting potential analogy with the known structure of *Lsamp* gene (Pimenta and Levitt, 2004). For a better reference, we discriminate between two parts in exon 1b (Figure 1A): 1b' (first part, specifically added to 1b transcripts) and 1b” (second part of exon 1b, which is included into all isoforms of *Opcml, Ntm, Lsamp* transcripts). Analogous gene structure was confirmed by respective alignment of *Opcml, Ntm*, and *Lsamp* 1a and 1b transcripts with Clustal Omega Multiple sequence alignment service (http://www.ebi.ac.uk/Tools/msa/clustalo/). Alternative transcripts were further confirmed by mapping representative EST sequences to both transcripts. A more detailed analysis of rodent and human transcripts was carried out by blasting potential I exon and II exon or their combination sequences against EST database. High quality ESTs spanning upstream of I or II exon were subsequently aligned to confirm the existence or lack of alternative first exons. Potential transcripts were confirmed by BLAST search in NCBI Refseq and Non-Refseq databases (Altschul et al., 1990). MEGA5 software was used to align EST sequences to transcripts (Tamura et al., 2011). *Opcml* and *Ntm* 1a promoter specific transcripts for rat were not available in NCBI nor Ensembl databases (accessed at 20/06/16) and were therefore amplified from the cDNA pool derived from the brains of male Sprague-Dawley rats and confirmed by sequencing. The alignment of *Opcml* and *Ntm* 1a promoter specific transcripts for rat, along with respective sequences from human and mice have been shown in Figure 1B. Other sequences used in the current study were obtained from respective databases in Ensembl (release 85) or NCBI. The genomic structure of *Opcml* and *Ntm* was confirmed by sequencing alternative 5′-regions of the transcripts presented in Figure 1B. The sequence identity numbers obtained from Ensembl database and accession codes available in NCBI RefSeq/Non-RefSec databases referring 1a and 1b isoform specific sequences have been shown in Supplementary Tables S1, S2.

**Figure 2:**
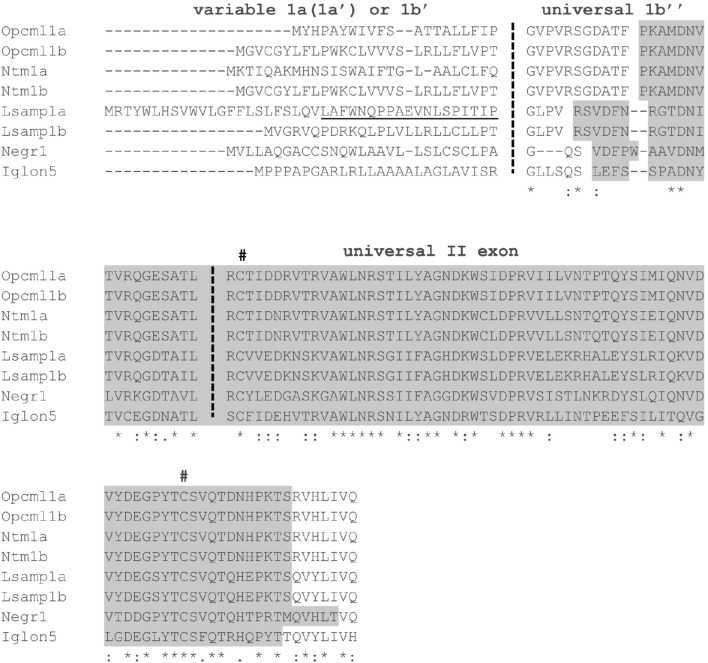
**Alignment of first and second exon specific amino acid sequences encoded by 8 known IgLON transcripts in mouse**. 1a or first part of exon 1b (1b') is separated by vertical dashed line from universally added exon 1b”. Underlined sequence indicates extra exon 1a' of *Lsamp* 1a transcript. First exons 1a (1a') or 1b' code for signal peptides. 1b” is separated by second dashed line from universally added exon 2. Exons 1b” and 2 code for first immunoglobulin domain indicated by box. # Indicates two conserved cysteine residues in the first Ig domain.

Conservation analysis was carried out to emphasize *Iglon5* (Refseq NP_001157990/Uniprot Q8HW98) affiliation with the IgLON family. The protein sequences for all IgLON isoforms cited in Supplementary Table S3 were aligned in Supplementary Figure S4. To highlight the 5′ region structure of IgLON proteins, the amino-acid alignment of variable (1a/1b') and universal (1b” ± II exon) parts of the proteins was performed (Figure 2). The amino-acid sequence identity between functional regions in IgLON family have been shown in Supplementary Tables S5–S9. The N-terminal signal peptide region, immunoglobulin domain sequences, and conserved cysteine residue locations were acquired from Uniprot. PredGPI web service was used to predict the location of GPI anchor binding sites (Pierleoni et al., 2008). All the alignments of either DNA and amino-acid sequences were performed by using the Clustal Omega Multiple sequence alignment program (Sievers et al., 2011).

### Animals

Detailed description of the creation of *Lsamp*-deficient mice with a LacZ transgene has been described by Innos et al. (2011). *Ntm* gene heterozygous mutant strain (032496-UCD B6;129S5-*Ntm*^*tm1Lex*^/Mmucd) were procured from the Mutant Mouse Regional Resource Center at UC Davis (https://www.mmrrc.org/catalog/sds.php?mmrrc_id = 32496) as described in Mazitov et al. (2017). The strategy for creation of *Ntm*-deficient mice was analogous to that of *Lsamp*-deficient mice as the exon 1b has been deleted, leading to the disruption of all functional *Ntm* transcripts. For obtaining *Lsamp*^−/−^, *Ntm*^−/−^, *Lsamp*^−/−^/*Ntm*^−/−^, and Wt mice for compensatory studies, we used the following scheme: first we created *Lsamp*^+/−^/*Ntm*^+/−^ animals by crossing *Lsamp*^−/−^ and *Ntm*^−/−^ mice; followed by crossing double heterozygous mice providing the necessary genotypes. C57BL/6 Bkl mice (ScanburAB, Sollentuna, Sweden) were used for collecting tissue samples from brain and other organs to describe the expression of IgLONs in wild-type mouse. The cDNA pool from the brain of Male Sprague-Dawley rats was used for sequencing *Opcml* and *Ntm* 1a 5′ regions for rat (Figure 1A). Breeding and housing was performed in the Institute of Biomedicine and Translational Medicine, University of Tartu. Mice were housed in 1264C Eurostandard type II cages (Tecniplast) measuring 268 × 215 × 141 mm. Cages contained aspen chips for bedding and aspen wool for nesting material which was replaced once a week. Each cage contained 7–8 animals based on allocation after weaning. The animals were maintained under 12-h light/dark cycle, with lights on at 7:00 a.m. and they had *ad libitum* access to food and water. All animal procedures in this study were performed in accordance with the European Communities Directive (86/609/EEC) and permit (No. 29, April 28, 2014) from the Estonian National Board of Animal Experiments.

### Tissue collection

The dissection of mouse brain was performed according to coordinates obtained from the mouse brain atlas (Franklin and Paxinos, 1997). Full mouse brain was divided into 17 areas to enable optimal brain-wide coverage. The areas dissected from the brain were as follows: areas of the cerebral cortex (frontal, parietal, occipital, and temporal cortex, the latter also including amygdaloid complex), the basal forebrain structures (caudate putamen, ventral striatum, septum pellucidum, hippocampus), the structures of the diencephalon (thalamus, hypothalamus, and pituitary gland), midbrain (including colliculi), pons, cerebellum, medulla oblongata, and spinal cord. Eyes with optic tract were also included in the list of neural tissues. We also dissected non-neural tissues including skeletal muscle, heart, lung, liver, small intestine, adrenal glands, kidney, male ureter, ductus deferens, testis, ovary, and uterus. At least three biological replicates (*n* ≥ 3) from each tissue were separated for the estimation. The tissue samples were individually dissected, immediately frozen in liquid nitrogen after dissection and stored in the −80°C freezer until further analysis (qPCR or western blot).

### Two-step qRT-PCR (qPCR)

IgLON transcript levels were determined by two-step RT-qPCR (qPCR). Total RNA was extracted from each tissue sample by the use of Trizol reagent (Invitrogen) according to the manufacturer's protocol. First strand cDNA was synthesized by the use of Random Hexamer (Applied Biosystems) and SuperScript^TM^ III Reverse Transcriptase (Invitrogen) according to the manufacturer's protocol. Quantitative TaqMan Assay with FAM-BHQ-probe was designed for the detection of *Opcml* 1a/1b, *Ntm* 1a/1b, *Negr1*, and *Iglon5* transcripts. *Lsamp* 1a/1b were detected as described previously in Philips et al. (2015). In case of *Opcml, Ntm*, and *Lsamp* universal reverse primer was combined with an alternative forward oligo specific for either 1a or 1b transcript. The approximate positions of primers used for amplification have been shown as bold arrows in Figure 1A. Custom TaqMan Probe was designed to bind to the universal 1b″ exon. *Synaptophysin* mRNA levels were determined by using the predesigned Taqman Gene Expression Assays (Applied Biosystems): Mm00436850_m1 (previously used also in Heinla et al., 2015). TaqMan Universal PCR Master Mix was used according to the manufacturer's protocol as reaction buffer. Two microgram of RNA was used in 20 μl end reaction for cDNA synthesis from brain tissues. Three microgram of RNA was used in 20 μl end reaction for cDNA synthesis from organ tissues. cDNA was diluted three times before 2.15 μl of cDNA was added per 42.3 μl reaction mix. Each reaction mix was divided out in 10 μl quadruplicates. ABI Prism 7900HT Sequence Detection System with ABI Prism 7900 SDS 2.4.2 software (Applied Biosystems) was used for qPCR detection.

### Protein extraction and western blotting

Eppendorf tubes containing the dissected mouse frontal cortex were snap-frozen in liquid nitrogen and stored at −80°C until use. Frozen tissues were sonicated in ice cold RIPA buffer (ThermoFisher Scientific) supplemented with protease inhibitor (Life Technologies). Protein lysates were centrifuged for 10 min 12,000 g at 4°C. Supernatant was collected and protein concentration was determined by BCA method (Pierce BCA Protein Assay Kit, Thermo Scientific). Samples were frozen at −80°C for long-term storage. NuPAGE Electrophoresis System (Life Technologies) components and equipment were used according to the manufacturer's instructions. For western blotting, membranes were blocked in 3% dry milk on a rocker for 60 min at RT. Next, membranes were incubated with mouse anti-Negr1 (1:200) (sc-393293, Santa Cruz), mouse anti-Ntm (1:200) (sc-390941, Santa Cruz), or rabbit anti-GAPDH (1:10K) (247002, Synaptic Systems) primary antibodies 60 min at RT and thereafter o/n at 4°C under gentle agitation. All primary antibodies were diluted in 3% dry milk with 0.1% Tween-20. After primary antibody incubation the membranes were washed six times in Milli-Q water and incubated with corresponding secondary antibodies for 1 h at RT under agitation. Secondary antibodies goat anti-mouse antibody (A21057, Invitrogen) and goat anti-rabbit antibody (35569, Jackson ImmunoResearch) were diluted in PBS-0.1% Tween-20 1:15 K and 1:40 K, respectively. After incubation with secondary antibodies the membranes were washed six times in Milli-Q water followed by 20-min wash with PBS-0.1% Tween-20 with shaking. Re-Blot Plus Strong Solution (1x) (2504, Millipore) was used to strip and re-use membrane. Antibody detection was performed using the LI-COR Odyssey CLx system (LI-COR Biotechnologies). Images were converted to grayscale and quantification was performed using Image Studio Lite v 3.1.4 (LI-COR Biotechnologies). Relative protein expression levels for NEGR1 and NTM were obtained after normalization to GAPDH.

### Data analysis and presentation

**Figure 3:**
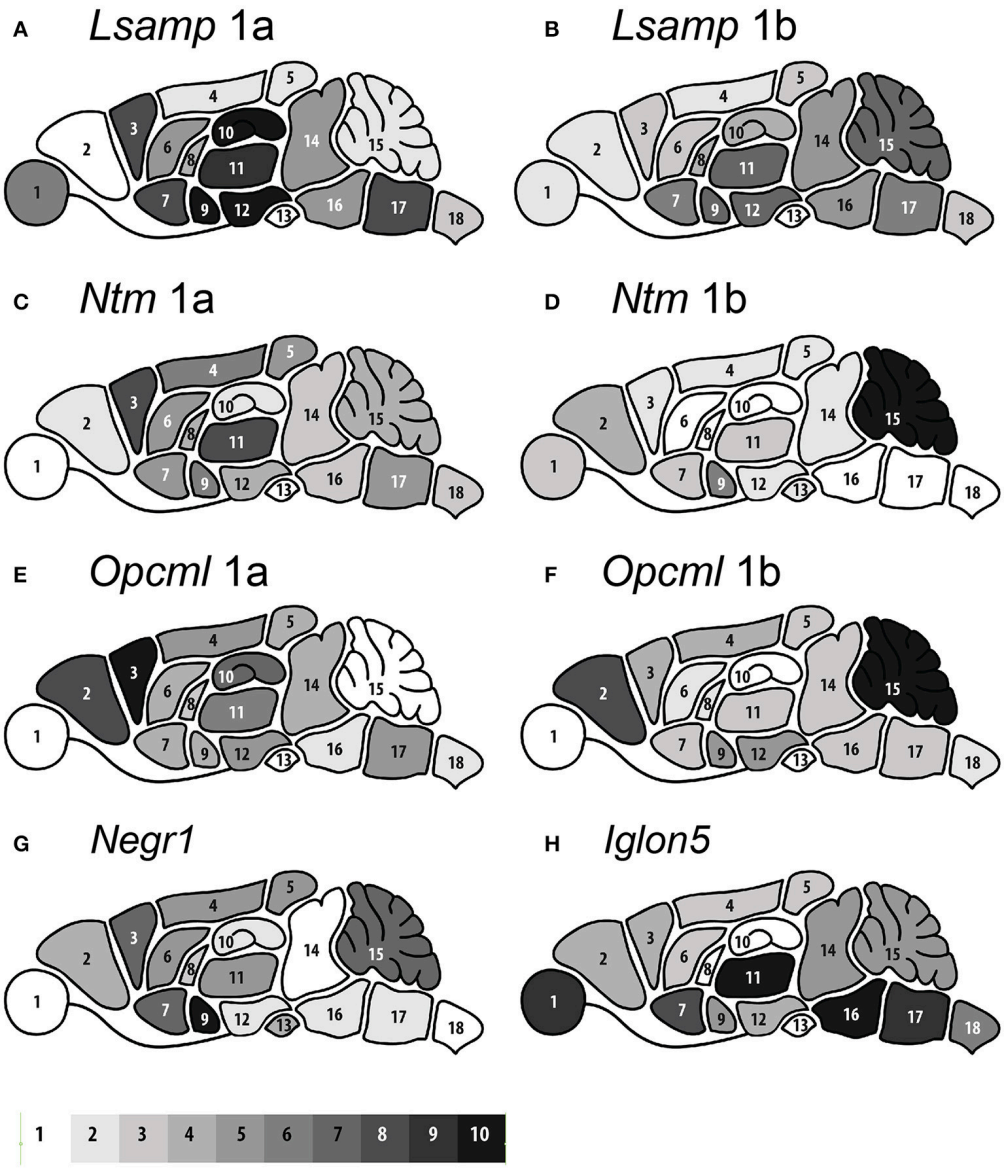
**The brain-wide expression distribution of eight IgLON transcripts based on relative expression intensity measured by qPCR. (A)** *Lsamp* 1a, **(B)** *Lsamp* 1b, **(C)** *Ntm* 1a, **(D)** *Ntm* 1b, **(E)** *Opcml* 1a, **(F)** *Opcml* 1b, **(G)** *Negr1*, and **(H)** *IgLon5*. The numbers on the mouse brain image (1–18) represent regions: eye (1), olfactory bulb (2), frontal cortex (3), parietal cortex (4), occipital cortex (5), temporal cortex including amygdaloid complex (9), caudate putamen (6), ventral striatum (7), septum pellucidum (8), hippocampus (10), thalamus (11), hypothalamus (12), pituitary gland (13), midbrain including colliculi (14), cerebellum (15), pons (16), medulla oblongata (17), and spinal cord (18). The 10-level gray-scale shades from white to black represent minimum (1–10%) to maximum (90–100%) expression levels respectively. The range of 10 intervals was achieved by dividing the difference of 2^−ΔCT^ minimum to maximum values separately for each gene. The exact 2^−ΔCT^ values (±SEM) for all qPCR data in the brain and non-neural tissues have been presented in Supplementary Table S10.

All qPCR data has been presented in the linear scale, in form of 2^−ΔCT^ (Livak and Schmittgen, 2001) where ΔCT is the difference in cycle threshold (CT) between the gene of interest (FAM) and housekeeper gene *Hprt1* (VIC). The use of internal control gene, *Hprt1*, has been carefully optimized in our previous studies (Raud et al., 2009) and has been proved to be one of the most stable housekeeping gene across different organs and tissue types also in other studies (Svingen et al., 2015). The exact 2^−ΔCT^ values (± *SEM*) in the brain and non-neural tissues have been presented in Supplementary Tables S10, S11; illustrative histograms depict expression data for selected tissues in Supplementary Figure S12. The brain-wide relative distribution of the expression levels of IgLON transcripts have been visualized in Figure 3. The numerical difference between minimum and maximum values of 2^−ΔCT^ was defined as expression range for each gene. The expression range was then divided into 10 ranks and converted to 10 grayscale shades from white to black, representing minimal (1–10%) to maximal (90–100%) intensity accordingly. The relative expression levels for each IgLON transcript in the mouse non-neural tissues has been presented in Table 1. The exact numerical values (mean 2^−ΔCT^ SEM) are available in the Supplementary Table S11. For a better overview, the expression levels detected by qPCR were categorized as firm (+ + +; the mean 2^−ΔCT^ ≥0.1), modest (++; the mean 2^−ΔCT^ ≥0.01), and minimal (+; the mean 2^−ΔCT^ ≥0.001). The estimations were given if the transcript specific amplicon was detected in at least three biological replicates and further corrected according to the quality of the amplification curve (Sisti et al., 2010).

**Table 1:** **The expression of IgLON transcripts in mouse non-neural tissues**.

	***Lsamp***		***Ntm***		***Opcml***		***Negr1***	***Iglon5***
	**1A**	**1B**	**1A**	**1B**	**1A**	**1B**		
Skeletal muscle		++			+++		+++	+++
Heart		+++	+	++		+	++	++
Lung					+		++	+
Liver	+	+		+	+		++	
Small intestine		+			+	+	+	+
Adrenal glands	++	++	+	+	+	+	+++	+
Kidney	+	+	++	+	+	+	++	++
Male ureter	+	+			+	+	+	+
Ductus deferens	++	+		+	+	+	++	+
Testis	+	+	+	+	+	+	++	+++
Ovary	+	++	++	+	++		++	++
Uterus	+	+	+	+	+	+	++	+

*The expression levels detected by quantitative real-time PCR were evaluated as firm (+ + +), modest (++), and minimal (+). The conversion criteria have been given in the “Methods” Section*.

### Statistical analysis of gene and protein expression data in mutant mice

**Figure 4:**
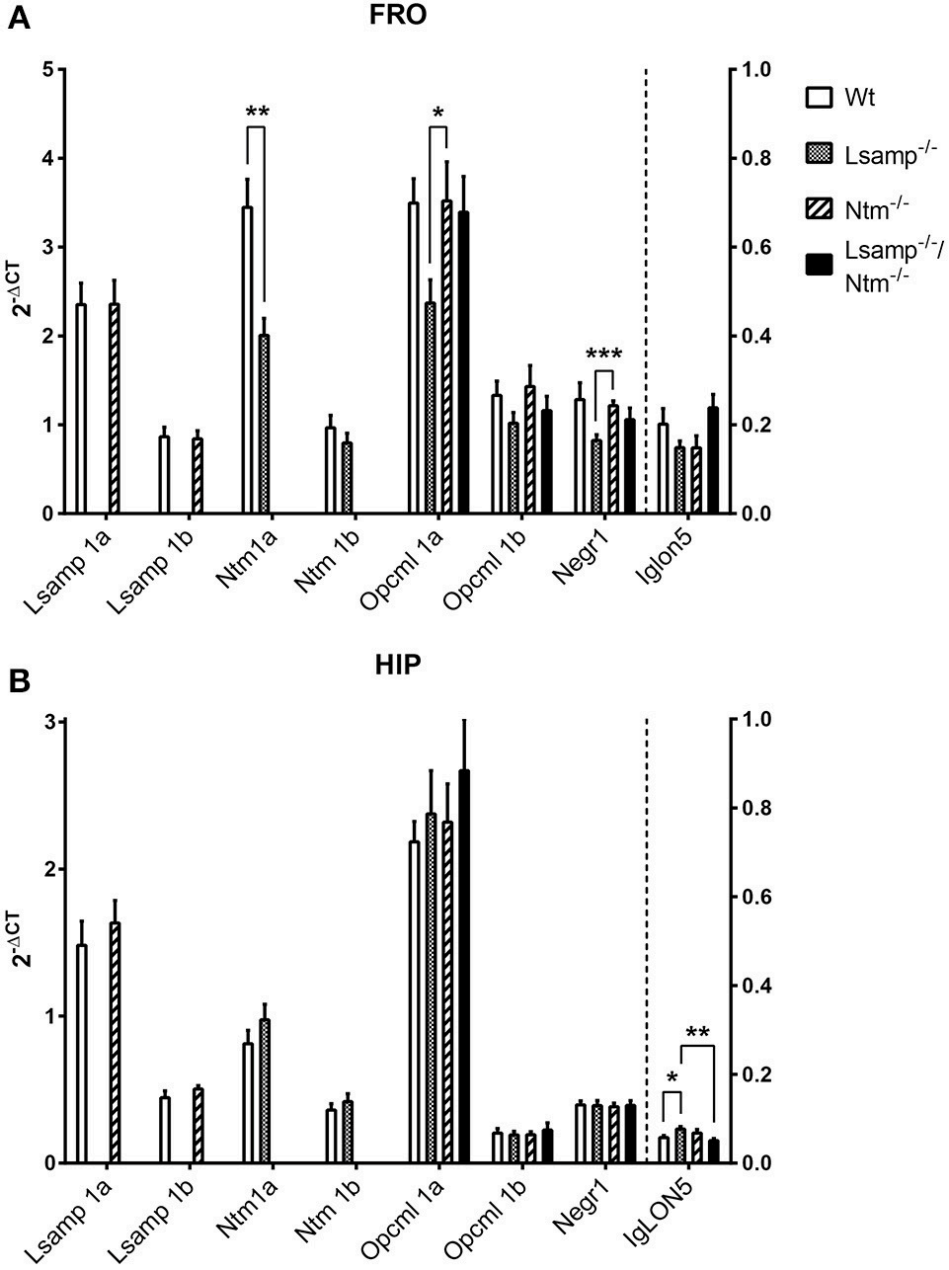
**The average mRNA expression level of IgLON transcripts in Wt, ***Lsamp***^**−/−**^, ***Ntm***^**−/−**^, and ***Lsamp***^**−/−**^/***Ntm***^**−/−**^ mutant mice measured by qPCR**. Expression levels are presented for **(A)** frontal cortex (FRO) and **(B)** hippocampus (HIP) based on promoter activity at the age of 4–5 months as 2^−ΔCT^ ± SEM. For *Opcml* 1a/1b, *Ntm* 1a/1b, *Lsamp* 1a/1b and *Negr1* the left Y-axis applies and for *Iglon5* (separated in the Figure by a vertical dashed line from the other transcripts) the right Y-axis applies. *n* = 6 in all groups; ^*^p < 0.05; ^**^p < 0.01; ^***^p < 0.001. One-way ANOVA was used for every isoform in parallel in two mutant subgroups: Group I: Wt (wild-type), *Lsamp*^−/−^, *Lsamp*^−/−^/*Ntm*^−/−^, and Group II: Wt, *Ntm*^−/−^, *Lsamp*^−/−^/*Ntm*^−/−^. Intra-group differences were further emphasized by Tukey's HSD test. Two mutant groups *Lsamp*^−/−^and *Ntm*^−/−^ were further compared by Student's *t*-test at significance level of α = 0.05. In the frontal cortex, the expression level of *Ntm 1a* was lower in *Lsamp*^−/−^ animals compared to Wt. The expression level of *Opcml 1a* and *Negr1* was lower in *Lsamp*^−/−^ animals compared to *Ntm*^−/−^. However, in the hippocampus, the level of *Iglon5* was significantly higher in *Lsamp*^−/−^ animals compared to both Wt and *Lsamp*^−/−^/*Ntm*^−/−^ groups. More detailed information about statistical findings can be found in the Sections Gene Expression Alterations in Frontal Cortex and Gene Expression Alterations in Hippocampus.

**Figure 5:**
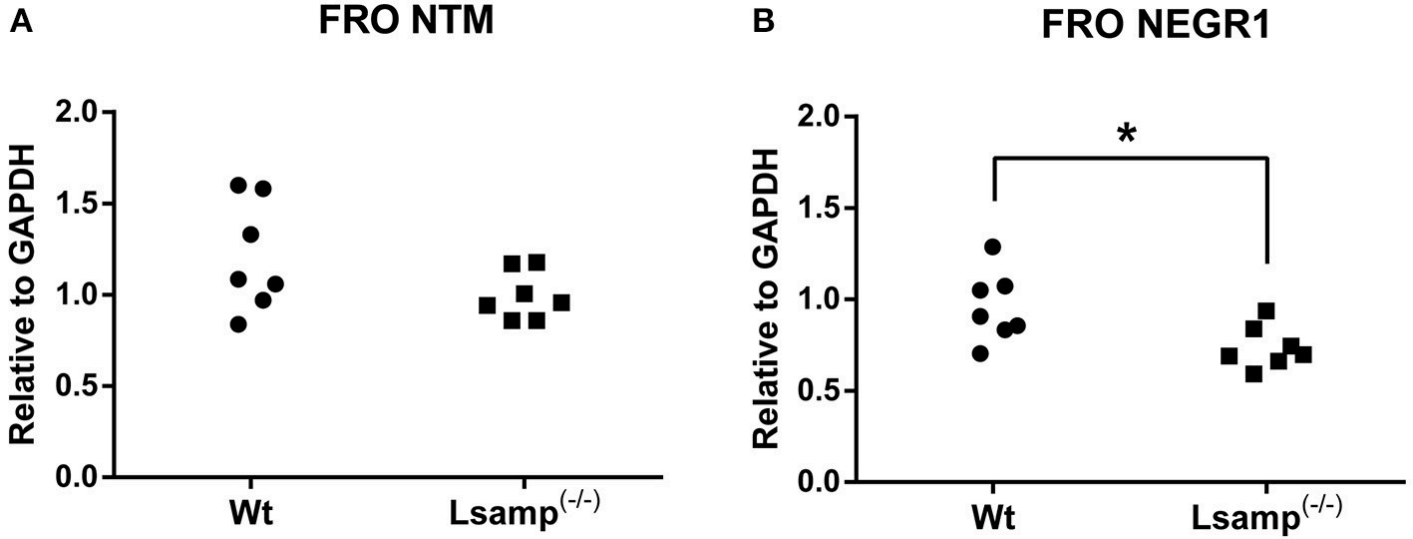
**The individual protein expression levels of NTM and NEGR1 in the frontal cortex of wild-type and ***Lsamp***^**−/−**^ mice measured by western blot. (A)** NTM and **(B)** NEGR1 expression levels are presented relative to GAPDH; *n* = 7; ^*^p < 0.05. It is important to note that NTM antibody is not able to discriminate between 1a and 1b isoforms, therefore the significant decrease of *Ntm* 1a in *Lsamp*^−/−^ animals (Figure 4A) is only detectable in mRNA level.

**Figure 6:**
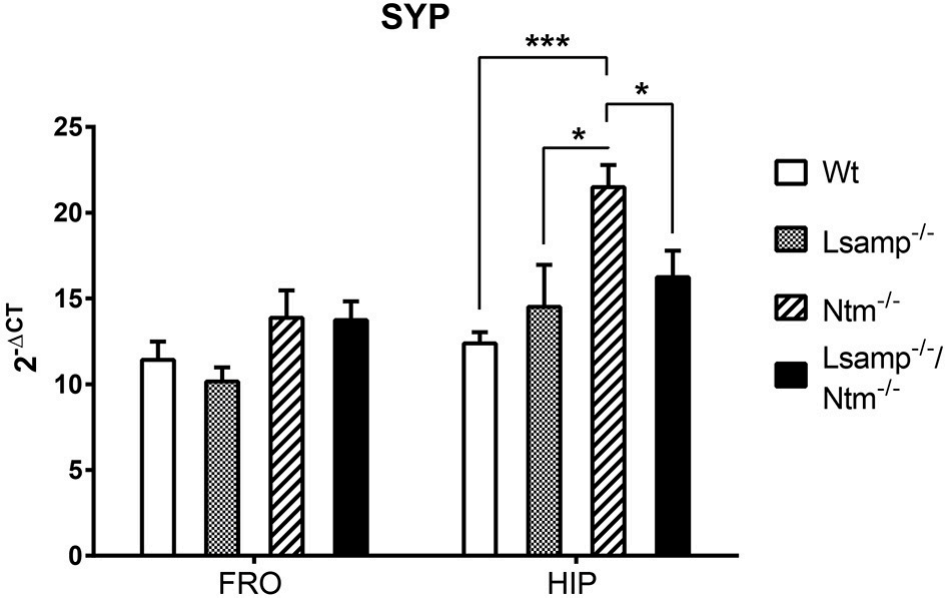
**The average mRNA expression level of ***Syp*** transcript in Wt, ***Lsamp***^**−/−**^, ***Ntm***^**−/−**^, and ***Lsamp***^**−/−**^/***Ntm***^**−/−**^ mutant mice measured by qPCR**. Expression levels are presented for frontal cortex (FRO) and hippocampus (HIP) based on promoter activity as 2^−ΔCT^ ± SEM. *n* = 6 in all groups; ^*^p < 0.05; ^***^p < 0.001. One-way ANOVA was used for every isoform in parallel in two mutant subgroups: Group I: Wt (wild-type), *Lsamp*^−/−^, *Lsamp*^−/−^/*Ntm*^−/−^, and Group II: Wt, *Ntm*^−/−^, *Lsamp*^−/−^/*Ntm*^−/−^. Intra-group differences were further emphasized by Tukey's HSD test. Two mutant groups *Lsamp*^−/−^and *Ntm*^−/−^ were further compared by Student's *t*-test at significance level of α = 0.05. Statistically significant differences in *Syp* expression were observed only in the hippocampus where the level was higher in *Ntm*^−/−^ animals than in Wt, *Lsamp*^−/−^/*Ntm*^−/−^, and *Lsamp*^−/−^ animals. More detailed information about statistical findings about *Syp* expression can be found in the Section Gene Expression Alterations in Hippocampus.

Alterations of IgLON gene expression levels in the frontal cortex and hippocampus of wild-type and mutant mice (Figures 4, **6**) were analyzed by one-way ANOVA in parallel in two mutant subgroups (Supplementary table S13): Group I included *Wt* (wild-type), *Lsamp*^−/−^, *Lsamp*^−/−^/*Ntm*^−/−^, and Group II included *Wt, Ntm*^−/−^, *Lsamp*^−/−^/*Ntm*^−/−^ at significance level of α = 0.05. Intra-group differences were further emphasized in separate mutant groups by Tukey's HSD-test carried out on significance level of α = 0.05. Two mutant groups, *Lsamp*^−/−^ and *Ntm*^−/−^, were further compared by Student's *t*-test at significance level of α = 0.05. In Western blot data analysis of relative protein expression levels for NTM and NEGR1 were normalized to GAPDH (Supplementary Figures S16, S17) and Student's *t*-test was used to detected statistically significant differences at significance level of α = 0.05 (Figure 5). Wild-type correlation analysis of IgLON expression levels in the frontal cortex and hippocampus was carried out with parametric Pearson correlation test at significance level of α = 0.05 to emphasize coherent expression within the family (Supplementary Figures S18, S19). The Shapiro-Wilks *W-*test preceded all gene and protein expression analysis to test for the normality assumption of the data using significance level of α = 0.05.

## Results

### *In silico* analysis of Iglon transcripts and proteins

*In silico* analysis of alternative first exons confirmed the existence of twin promoter structure for *Opcml, Ntm*, and *Lsamp* genes in rodent and human. The schematic representation of twin promoter structure of *Opcml, Ntm*, and *Lsamp* genes has been shown in Figure 1A. The alignments of unique promoter specific 5′ sequence fragments of transcripts can be viewed in Figure 1B and alignment of amino-acid sequences of 5′ region in IgLONs have been presented in Figure 2. Different isoforms and potential variants of exon structure of IgLON family transcripts based on Ensembl.org database can be viewed in Supplementary Tables S1, S2. Available EST sequences and blast searches also support the existence of both promoter specific transcripts for all three genes. *Negr1* and *Iglon5* in mouse have a uniform 5′ end of the transcript, suggesting single promoter. The number of alternative variants in the 3′ region of IgLON transcripts is remarkable, for example, the mouse *Negr1* gene is confirmed to have two alternative transcripts based on EST alignments, both of which have alternative 3′ ends (NM_001039094/NM_177274; Supplementary Table S3). However, the variation in the 3′ end region was not in the focus of the current study and will be addressed separately in the future. *Iglon5*, the most recently discovered family member, has currently support for only one transcript for rodent and human based on EST data, indicating single promotor. IGLON5 spans 336 amino acids and is on the protein level closest to OPCML (50%), NTM (48–49%), and LSAMP (46–47%), and least similar to NEGR1 (41%). IgLON5, as all IgLONs, has N-terminal signal peptide, three pairs of cysteine residues and three C2 immunoglobulin domains (Supplementary Figure S4). Cysteine residues are highly conserved as expected as they form disulfide bridges to stabilize immunoglobulin domain structure. GPI prediction service indicated all IgLON transcripts to have putative GPI binding site. OPCML 1a/1b, NTM 1a/1b, and IGLON5 have conserved residue position (N) for GPI binding. Alternative 3′ end isoforms of LSAMP (1a/1b) and NEGR1 (A0A4W9/Q80Z24) have alternative GPI binding sites (Supplementary Figure S4). Average overall similarity of IgLONs varies from as high as 72% between OPCML and NTM to as low as 41% between NEGR1 and IGLON5 (Supplementary Table S5). Overall conservation is the highest in immunoglobulin domains (Supplementary Tables S7–S9) and the lowest in the area coding for signal peptide (Supplementary Table S6). Comparison of domains reveals the first two immunoglobulin domains to be more conserved. Conservation varies from as high as 91% between OPCML and NTM to as low as 42% between NEGR1 and IGLON5 in domain I, 71% between OPCML and NTM to as low as 47% between NEGR1 and IGLON5 in domain II and 74% between OPCML and NTM to as low as 45% between NEGR1 and IGLON5 in domain III.

### The expression of IgLON transcripts in the mouse brain

The brain-wide distribution of all IgLON genes is highly heterogeneous across the anatomical regions and across individual transcripts. The grayscale illustrations (Figures 3A–H) represent relative expression pattern within each gene including its alternative transcripts: all IgLON genes with alternative promoters 1a and 1b (Figures 3A–F) reveal remarkable dissimilarity in the activity levels of two promoters in different brain areas just as we have previously demonstrated for *Lsamp* in Philips et al. (2015). *Lsamp* 1a (Figure 3A) is more intensively expressed in the hippocampal area than *Lsamp* 1b (Figure 3B), on the contrary, the expression of *Lsamp* 1b is more intense in the cerebellum, compared with *Lsamp* 1a transcript. Likewise, the general expression patterns of *Lsamp* transcripts are in line with our previous studies (Heinla et al., 2015; Philips et al., 2015). As a novel finding, *Lsamp* 1a promoter has strong activity in the eye (Figure 3A).

The expression activity of *Ntm* 1a and 1b promoters is remarkably different. *Ntm* 1a promoter activity is the highest in the frontal cortex and thalamus (Figure 3C), whereas 1b is the most active in the cerebellum (Figure 3D). Likewise, the expressional activity of *Opcml* 1a and 1b promoters is highly dissimilar. The highest expression of *Opcml* 1a was detected in the frontal cortex (Figure 3E), whereas the highest expression of *Opcml* 1b was measured in the cerebellum (Figure 3F) where 1a promoter has no detectable activity. However, both promoters in *Opcml* are highly active in the olfactory bulb. *Negr1* has the most intense expression levels in the temporal cortex including amygdaloid complex and relatively high expression in the cerebellum, cerebral cortex, and basal forebrain. The signal of *Negr1* transcript was minimal in the brainstem (midbrain, pons, medulla, spinal cord) and in the eye (Figure 3G). The level of *Iglon5* transcript, on the contrary, was highest in the brainstem (namely pons and medulla), thalamus and eye and weakest in the hippocampus, septum, and pituitary gland (Figure 3H; Supplementary Table S10).

### The expression of IgLON transcripts outside the central nervous system in mouse

With a few exceptions, IgLON transcripts could be detected in all non-neural tissues that we studied: in the skeletal muscles, heart, lung, liver, small intestine, adrenal glands, kidney, male ureter, ductus deferens, testis, ovary, and uterus (see Table 1 for overview and Supplementary Table S11 for exact expression values represented in the units of 2^−ΔCT^).

*Lsamp* 1a and 1b transcripts have detectable expression across the urogenital system. Promoter specific expression (only 1b promoter is expressed) can be seen in the heart, skeletal muscle, and small intestine, which all consist specific types of muscle cells. The activity of both *Lsamp* promoters was detectable in the liver but there was no signal in the lungs. The expression of *Ntm* is seemingly more selective. The signal for both promoters was missing in the lungs, small intestine, male ureter, and skeletal muscle. Both 1a and 1b promoters of *Ntm* gene are active in the heart, kidneys, adrenal glands, testis, ovary, and uterus. 1b transcript specific signal was specifically detectable in the liver and *ductus deferens*. *Opcml* mRNA specific signals were detectable in all organs/tissue types studied; *Opcml* 1a promoter was specifically active in the skeletal muscle, lungs, liver, and ovaries; only *Opcml* 1b promoter was active in the heart. The expression activity of *Negr1* was detectable in all organs/tissue types studied. *Iglon5* mRNA was detectable in all other tissues except liver.

Relative quantification was also used for the analysis of non-neural tissues as our purpose was to gain insight to the relative expression of IgLON transcripts in non-neural tissues compared with the expression levels in the neural tissues. All IgLONs have remarkably higher expression levels in the neural tissues compared to any other non-neural tissue type. However, the average fold-difference (average expression in the brain/average expression in non-neural tissues; across all samples) between expression in the brain and organs was different in IgLON transcripts. The most homogenous body-wide anatomical distribution was characteristic for *Iglon5*, as the average expression in the brain was roughly only 4.5-fold higher than the average *Iglon5* mRNA expression level in the non-neural tissues. The expression difference (neural vs. non-neural) was also lower for *Negr1* (14.4-fold) and *Lsamp* 1b (16.6-fold) transcripts. The other IgLON transcripts had considerably high difference between the expression in the brain and the expression in non-neural tissues (58.8-fold for *Lsamp* 1a, 51.7-fold for *Ntm* 1a, 55.5-fold for *Ntm* 1b; 61.3-fold for *Opcml* 1a, 93.7-fold for *Opcml* 1b).

### Gene expression alterations in the brain of wild-type and mutant mice

#### Gene expression alterations in frontal cortex

The expression values (mean 2^−ΔCT^ ± *SEM*) for the IgLON transcripts in the frontal cortex are presented in Supplementary Table S14 and Figure 4A Expression alterations were detected for *Opcml* 1a, *Ntm 1a*, and *Negr1*. A statistically significant difference was observed in Group I [*F*_(2, 15)_ = 3.81, one-way ANOVA *p* = 0.046]. Further intra-group analysis did not reach statistical significance between Wt and *Lsamp*^−/−^ (Tukey HSD *p* = 0.06), nor between *Lsamp*^−/−^ and *Lsamp*^−/−^/*Ntm*^−/−^ (Tukey HSD *p* = 0.092). A statistically significant difference was detected for *Opcml 1a* in comparison of different mutant lines *Lsamp*^−/−^ and *Ntm*^−/−^ [*F*_(2, 10)_ = 2.85, *t*-test *p* = 0.049]. The average expression level of *Ntm 1a* transcript in the frontal cortex was significantly reduced in *Lsamp*^−/−^ compared to Wt [*F*_(2, 10)_ = 2.66, *t*-test *p* = 0.003]. The average expression level of *Negr1* transcript in the frontal cortex was reduced in *Lsamp*^−/−^ compared to *Ntm*^−/−^ [*F*_(2, 10)_ = 1.41, *T*-test *p* = 0.001; Figure 4A]. There were no statistically significant effects in the expression of *Syp* in the comparison of the frontal cortex of Wt and different mutant lines (Figure 6).

#### Gene expression alterations in hippocampus

The expression values (mean 2^−ΔCT^ ± SEM) for the IgLON transcripts in the hippocampus are presented in Supplementary table S15 and Figure 4B. Expression changes were detected for *Iglon5* and *Syp*. For *Iglon5* transcript in the hippocampus a statistically significant difference was observed in Group I [*F*_(2, 15)_ = 6.8, one-way ANOVA *p* = 0.008]. Further intra-group analysis revealed a significantly higher expression level in the *Lsamp*^−/−^ group as compared to Wt (Tukey HSD *p* = 0.045) and *Lsamp*^−/−^/*Ntm*^−/−^ (Tukey HSD *p* = 0.008; Figure 4B). The expression levels of *Syp* in the hippocampus were significantly different in Group II [*F*_(2, 15)_ = 13.99, one-way ANOVA *p* = 0.0004]. Further intra-group analysis showed that the expression was significantly higher in the *Ntm*^−/−^ group as compared to Wt (Tukey HSD *p* = 0.0004), *Lsamp*^−/−^/*Ntm*^−/−^ (Tukey HSD *p* = 0.022), and *Lsamp*^−/−^ [*F*_(2, 10)_ = 3.59, *t*-test *p* = 0.031; Figure 6].

#### Protein expression in the frontal cortex

Selected effects on mRNA level were thereafter explored at the protein level in the frontal cortex. NTM average protein expression measured by western blot was lower in *Lsamp*^−^^/−^ (1; *n* = 7) mice when compared with the Wt (1.21; *n* = 7) mice, however, this effect did not reach statistical significance [*F*_(2, 12)_ = 5.13, *t*-test *p* = 0.11; Figure 5A]. It is important to note that NTM antibody is not able to discriminate between 1a and 1b isoforms, therefore the significant decrease of *Ntm* 1a in *Lsamp*^−/−^ animals is only detectable in mRNA level. NEGR1 average protein expression was significantly lower in *Lsamp*^−^^/−^ (0.74; *n* = 7) mice when compared with Wt (0.96; *n* = 7) mice [*F*_(2, 12)_ = 2.74, *t*-test *p* = 0.023; Figure 5B].

## Discussion

### Structural characteristics of IgLONs

In the present study, we have performed a comprehensive promoter-specific analysis of the anatomical distribution of IgLON transcripts based on a thorough analysis of the genomic and expressed sequences of the whole IgLON family. According to our analysis *Ntm* and *Opcml* display similar genomic structures as *Lsamp* (Pimenta and Levitt, 2004) characterized by two alternative promotors 1a and 1b, whereas *Negr1* and *Iglon5* have a uniform 5′ end of the transcript, suggesting single promoter. The alternative 5′ region 1a/1b is encoding for alternative N-terminal signal peptides (Figure 2) which are most likely used to target nascent proteins to specific cellular region. Signal peptides are cleaved during post-translational modifications and the processed polypeptides contain identical N-termini, indicating that the complex alternative splicing process serves regulatory mechanisms without altering the functional properties of the protein (Pimenta and Levitt, 2004). Alternative promoters are considerable mechanisms by which genes obtain functional flexibility and diversity. For example, *Bdnf* gene in rodent and human has nine promoters, which enable tissue-specific and developmentally regulated expression of different isoforms. As in case of IgLONs, these *Bdnf* isoforms have been reported to code the same core part of protein, with alterations in signal peptide (Pruunsild et al., 2007). Having multiple cell or tissue specific promoters can provide opportunity for quick adaptation to a changed environment (Xin et al., 2008). Furthermore, according to our previous data, one of the IgLON protein, LSAMP, is indeed implicated in the plasticity and adaptability in changing environments (Innos et al., 2012; Heinla et al., 2015).

Based on our analysis, *Iglon5*, the recently characterized family member, shares high level of conservation with all other IgLON family members. Protein sequence level conservation ranks IGLON5 to be closest to OPCML, NTM, LSAMP, and least similar to NEGR1 (Supplementary Table S5). IGLON5 also shares structural qualities characteristic to IgLON protein family: N-terminal signal peptide, GPI anchor binding site and three pairs of cysteine residues for three immunoglobulin domains (Figure 2; Supplementary Figure S4). The first two immunoglobulin domains share the highest intra-family conservation. Extending distally from the cell membrane, it is likely that these domains are used for the formation of *cis* and *trans* interactions within IgLON family, analogously with the thoroughly described intra-family dimerization pattern of Nectins (Narita et al., 2011).

### Expression of IgLON transcripts outside the central nervous system in mouse

It has been emphasized for decades that IgLON adhesion molecules are specific for the central nervous system (CNS) (Horton and Levitt, 1988; Pimenta et al., 1995). Most of the conventional methods give no IgLON-specific signal in non-neural tissue samples. LSAMP specific signal has not been detected in the liver and kidney by using northern blot (Pimenta and Levitt, 2004) or western blot (Innos et al., 2011), although strong LSAMP specific signals were present for brain samples in the same immuno/ hybridization membrane. Likewise, NEGR1 specific signal was detectable only in the brain, but not in any of the non-neural tissues (lung, liver, kidney, testis, muscle) that were run on the same immunoblot (Funatsu et al., 1999). By using quantitative polymerase chain reaction (qPCR) that is widely acknowledged as the most sensitive method to quantify small amounts of nucleic acids (Svec et al., 2015), we detected the expression of both *Lsamp* 1a and 1b transcripts in the liver and kidney in the current study and *Negr1* transcript was detectable in all non-neural tissues that were tested. With a few exceptions, the IgLON transcripts could be detected in all tissues outside CNS that we studied: in the skeletal muscles, heart, lung, liver, small intestine, adrenal glands, kidney, male ureter, ductus deferens, testis, ovary, and uterus (Table 1). We have used relative quantification for the analysis of IgLON expression in both neural and non-neural tissues, aiming to evaluate the relative difference in expression levels in the brain and other organs. The expression difference between brain and other organs was highly different across IgLON genes ranging from 4.5-fold (*Iglon5* transcript) to nearly 100-fold (*Opcml* 1b transcript). This differences explain why IgLON proteins/transcripts often remain undetectable in non-neural tissues where the copy number of IgLON transcripts is low and not detectable by expression techniques with modest sensitivity, such as western blot and northern blot. *Iglon5* seems to be different in its relatively homogenous body-wide expressional distribution and is not characterized by obvious expressional dominance in the central nervous system which is a definite characteristic of other IgLON family members.

Outside CNS, the highest expression level was observed for *Lsamp* 1b transcript in the heart, which is in line with considerable evidence suggesting that IgLONs, specifically *Lsamp* and *Ntm*, may have additional functions in the human cardiovascular system (Wang et al., 2008; Luukkonen et al., 2012; Cao et al., 2015; Dungan et al., 2016). However, data from knockout mouse models has demonstrated that deletion of either *Lsamp, Ntm*, or *Opcml* individually, or *Opcml* and *Ntm* together as a double heterozygote, does not result in apparent cardiac phenotype (Innos et al., 2011; Ye et al., 2014).

The accumulating data indicating that IgLON neural adhesion molecules can function as tumor-suppressor genes in a number of organs also outside CNS (Sellar et al., 2003; Cui et al., 2008; Kim et al., 2014; Coccaro et al., 2015) has been rather unexpected as the expression-distribution has been suggested to be limited to CNS. Mutations or epigenetic alterations in the IgLON genes have been linked with different tumor types in the CNS (Reed et al., 2007; Minhas et al., 2013) but also in many other tissue types including osteosarcomas in the bone tissue (Barøy et al., 2014) and malignant processes involving blood cells, such as acute lymphoblastic leukemia (Kang et al., 2012) and acute myeloid leukemia (Coccaro et al., 2015). Majority of IgLON-related tumors in non-neural tissues involve urogenital system and reproductive system. Evidence links *OPCML* gene with epithelial ovarian cancer (Sellar et al., 2003; Ntougkos et al., 2005). Mutations in the *NEGR1* gene have been related to Wilms tumor, which leads to malignant process in the kidney (Karlsson et al., 2011), additionally *LSAMP* promoter has been inactivated by methylation in a considerable amount of clear cell renal carcinomas. One or multiple members of the IgLON family have also been altered in bladder cancer (Pascal et al., 2009), prostate cancer (Huang et al., 2013), uterine leiomyomata (Crabtree et al., 2009), and in the testicular cancer (Cheung et al., 2010). Here, we confirmed widespread expression of IgLON transcripts in the urogenital and reproductive system. Additionally, our data suggests IgLON adhesion molecules to mediate intercellular interactions also in non-neural tissues which is likely the mechanism for the tumor formation and invasion (Shimizu et al., 2016) often reported in case of disruptive mutations in IgLON genes.

### Expression of IgLON transcripts in the brain

Twin promoters 1a and 1b of *Opcml, Ntm*, and *Lsamp* (Figures 3A–F) reveal remarkable variety in the activity levels in different brain areas as we have previously demonstrated for *Lsamp* gene (Philips et al., 2015). Certain expression patterns can be detected between promoters: whereas 1b promoters are highly expressed in the cerebellum, 1a promoters, on the contrast, have stronger expression in the frontal cortex and in certain basal forebrain structures, such as dorsal striatum and hippocampus. The general expression patterns of *Lsamp* transcripts is in line with our previous studies (Heinla et al., 2015; Philips et al., 2015). As a novel finding, *Lsamp* 1a promoter has strong activity in the eye. The expressional activity of *Ntm* 1a and 1b promoters is remarkably different as well. 1a promoter activity is the highest in the frontal cortex and thalamus (Figure 3C), whereas 1b is the most active in the cerebellum (Figure 3D). In the light of these results, it appears that the high levels of *Ntm*, previously detected in the thalamus, basal ganglia, and dorsal striatum (Struyk et al., 1995), is triggered by *Ntm* 1a promoter, whereas the high expression of *Ntm* in the cerebellum, detected in the same study, is initiated from *Ntm* 1b promoter. Lower levels of *Ntm* in the brain stem nuclei and spinal cord (Struyk et al., 1995; Gil et al., 2002) represent *Ntm* 1a activity. *Ntm* 1a promoter activity was minimal in the brainstem in the current study.

Likewise, the expression activity of *Opcml* 1a and 1b promoters varies highly. Considering our current results, it appears that the high levels of *Opcml*, previously detected in the hippocampus of adult rat (Hachisuka et al., 2000), is triggered by *Opcml* 1a promoter. The highest expression of *Opcml* 1a was detected in the frontal cortex (Figure 3E), whereas the highest expression of *Opcml* 1b was measured in the cerebellum (Figure 3F) where 1a promoter has no detectable activity. However, both promoters in *Opcml* are highly active in the olfactory bulb. The expression of *Opcml* has been well characterized in the primary visual cortex of the cat (Li et al., 2006). The expression activity in the occipital lobe was mild and nearly equal for both 1a and 1b promoters in our current study. The high expression activity of *Negr1* in the frontotemporal cortex has been described previously by using *in situ* hybridization (Brauer et al., 2000) and highest levels of *Negr1* in the frontal and temporal cortex were also detected in the current study. Here, we demonstrate for the first time the expression profile of *Iglon5* transcript. The levels of *Iglon5* transcript were the highest in the thalamus and brainstem (namely pons and medulla); the expression activity was also relatively high in non-neural organs and tissue types, being roughly only 4.5-fold lower than the average *Iglon5* expression in brain areas.

### Intra-family interactions of IgLON family

Several cell culture experiments suggest LSAMP and other IgLON proteins to have vital functions in the development of neural circuits, as LSAMP has been shown to regulate the formation of septohippocampal (Keller et al., 1989), intrahippocampal (Pimenta et al., 1995), and thalamic (Mann et al., 1998) connections. Likewise, NTM has been shown to participate in the axon fasciculation of cerebellar systems, in the formation of excitatory synapses (Chen et al., 2001) and neurite outgrowth (Gil et al., 1998). Despite a seemingly essential function in brain development, no major changes have been reported in the gross anatomy in the brains of mice deficient for either *Lsamp* (Philips et al., 2015) or *Negr1* (Lee et al., 2012). Considering the high levels of intra-family homology shared by IgLON adhesion molecules and functional compensation observed between intra-family genes of other cell adhesion molecules (Giagtzoglou et al., 2009; Bedner et al., 2012) led us to investigate the potential compensatory effects in the level of IgLON transcripts/proteins in the absence of one or two family members.

Surprisingly, in the current study we found no increase of the transcripts of other IgLON family genes in the frontal cortex of mice lacking *Ntm, Lsamp*, or both the genes (Figure 4). On the contrary, we demonstrated significantly reduced expression of *Ntm* 1a mRNA and NEGR1 protein in *Lsamp*-deficient mice. *Negr1* transcript was significantly reduced in the frontal cortex of *Lsamp*-deficient mice compared with the frontal cortex of mice lacking *Ntm*. The double-mutant mice itself revealed no significant changes compared to wild-type mice. Moreover, we found the intra-family expressions of IgLON transcripts across individuals to be highly correlated (Supplementary Tables S18, S19). Strict maintenance of definite expression pattern of IgLONs in different brain areas or cell types can be necessary for normal function of neural circuits; loss of binding partner may lead to down-regulation of other family member to inhibit unbalanced dimer formation. Our current results suggest that IgLON adhesion molecules perform specific functions and lack of one family member is not compensated by the quantitative increase of the others. Rather, the family seems to function collectively in a balanced manner as suggested by previous studies demonstrating highly specific complexes and interactions between IgLON family members (Mann et al., 1998; Gil et al., 2002; Reed et al., 2004).

The reduced expression of other IgLON transcripts in *Lsamp*-deficient brain seems to be specific to certain brain areas or cell types: in our study the changes were detectable in the frontal cortex but not in the hippocampal area. In the hippocampus, the only significant effects were changes in the level of *Iglon5* expression in *Lsamp*-deficient hippocampi compared with hippocampi of wild type mice and mice lacking both *Ntm* and *Lsamp*. The result has no clear explanation as there is no data about the interactions of *Iglon5* with other IgLON family members. However, our results suggest that the intra-family interactions including potential compensatory effects can be tissue specific.

As previously published data links IgLON family members with synaptic plasticity (Hashimoto et al., 2009; Qiu et al., 2010; Heinla et al., 2015), we used our mutant mouse models also to explore the intra-family interactions acting on the expression levels of well-described marker of synaptogenesis, *Synaptophysin* (*Syp*). We detected significant upregulation of *Syp* mRNA in the hippocampi of mice lacking *Ntm*, but not in mice lacking *Lsamp* (Figure 6). The results suggest that *Ntm* could have an inhibitory impact on the reconstructive activity in the hippocampal synapses. Moreover, as *Syp* is not significantly increased in the hippocampi of mice lacking both *Ntm* and *Lsamp*, we can hypothesize that LSAMP is the real enhancer for processes that are correlated with *Syp* activity in the hippocampus. Indeed, overexpression of LSAMP in hippocampal cell culture has been shown to have a stimulating effect on synapse formation (Hashimoto et al., 2009). Additionally, overexpression of NTM in the same experiment had no effect on the synapse marker, supporting the notion of NTM as an inhibiting/neutral factor for synaptogenesis. Hashimoto et al. (2009) also overexpressed both NTM and LSAMP, which resulted in a decrease of synapses. Our data combined with previously published results suggest that NTM and LSAMP are involved in synaptogenesis, having antagonistic functions which could be characteristic for the whole IgLON family.

In conclusion, we show that important regulatory mechanisms of IgLON genes derive from their genomic organization. Remarkable variety in the activity levels of 1a and 1b promoters both in the brain and a wide spectrum of tissues outside the central nervous system, suggests a complex regulation of IgLONs by alternative signal peptides driven by 1a and 1b promoters. As the lack of one or two family members is not compensated by a systematic quantitative increase of the other members, IgLON proteins seem to have limited redundancy despite highly homologous sequence in both DNA and amino acid level. The antagonistic impact of individual IgLONs in functional systems is supported here by differential *Syp* expression in deletional mutants of IgLON members. Altogether our results emphasize the importance of studying IgLON family members as a complex functional system when interpreting their biological role.

## Author contributions

MP, KL, and EV planned the study. TVa performed *in silico* and statistical analysis. TVi, TVa, KL, and MP dissected tissue samples. MP designed the qPCR assay. TVa and JN performed qPCR, KE performed Western Blot. KS, KL, IH, MJ, and JI were involved in breeding and maintaining of mutant mice. MP and TVi wrote the paper. KS, TVa, IH, MJ, JI, KL, and EV participated in data interpretation and revision of the paper and all authors approved the final version.

## Funding

This study was supported by an institutional investigation grant from the Estonian Research Council IUT20-41 (EV) and personal investigation grant from the Estonian Research Council PUT129 (MP). This research was also supported by the European Union through the European Regional Development Fund (Project No. 2014-2020.4.01.15-0012). KE was supported by the Estonian Research Council grants PUT (120 and 1077).

### Conflict of interest statement

The authors declare that the research was conducted in the absence of any commercial or financial relationships that could be construed as a potential conflict of interest.
