# Supplementary material for: Promoter-Specific Expression and Genomic Structure of IgLON Family Genes in Mouse
Source: Front Neurosci. 2017 Feb 2;11:38. doi: 10.3389/fnins.2017.00038 (PMC5288359; doi:10.3389/fnins.2017.00038)

Supplementary Material

Promoter-specific expression and genomic structure of IgLON family genes in mouse

Taavi Vanaveski[1], **Katyayani Singh**[1], **Jane Narvik**[1], **Kattri-Liis Eskla**[1], **Tanel Visnapuu**[1,2], **Indrek Heinla**[1], **Mohan Jayaram**[1], **Jürgen Innos**[1], **Kersti Lilleväli**[1], **Mari-Anne Philips**[1*], **Eero Vasar**[1]

[1] Department of Physiology, Institute of Biomedicine and Translational Medicine, University of Tartu, Tartu, Estonia

[2] Division of Pharmacology and Pharmacotherapy, Faculty of Pharmacy, University of Helsinki, Helsinki, Finland

*** Correspondence:** Department of Physiology, Institute of Biomedicine and Translational Medicine, University of Tartu, Tartu, Estonia. E-mail: marianne.philips@ut.ee

Supplementary table S1. Sequence IDs/accession numbers of IgLON family transcripts characterized by twin promoter structure. IgLON transcript sequence IDs have been here designated as 1a/1b isoforms for the mouse, rat and human. The 1a/1b discrimination is not publicly available in NCBI (RefSeq and Non-RefSeq) and Ensemble.org databases

			Ensembl.org	NCBI	
**Species**	**Gene**	**Variant**		**RefSeq**	**Non-RefSeq**
Mus musculus	***Lsamp***	**1A**	NA (not available)	NA	AK164171.1 AK141903 AK053103
		**1B**	Lsamp-001 ENSMUST00000078873	NM_175548.3	AK140663 AK140043 AK158702 AK044845 AK030681
Homo sapiens	***LSAMP***	**1A**	LSAMP-201 ENST00000539563	NA	AK299851.1
		**1B**	LSAMP-001 ENST00000490035	NM_002338.3	BC033803 BC022345
Rattus norvegicus	***Lsamp***	**1A**	NA	NA	NA
		**1B**	ENSRNOT00000042024	NM_017242.1 XM_006248339	BC087607 AY326256
Mus musculus	***Ntm***	**1A**	Ntm-001, ENSMUST00000075069	NM_172290.3	AK140516.1 AK046377.1 AK045973.1 AK138263.1
		**1B**	Ntm-004, ENSMUST00000115237	XM_006510208 XM_006510207 XM_006510205	BC023307 AK158752 AF282980
Homo sapiens	***NTM***	**1A**	NTM-002 ENST00000374791	NM_001048209.1	AK315964.1 AK293487.1 AY358331.1 AK294137.1
		**1B**	NTM-001, ENST00000374786	NM_016522.2 NM_001144058.1 NM_001144059.1	AF126426.1
Rattus norvegicus	***Ntm***	**1A**	NA	NA	NA
		**1B**	ENSRNOG00000023720	NM_017354 XM_008766065 XM_006242749	
Mus musculus	***Opcml***	**1A**	Obcml-001 ENSMUST00000115243	NM_177906.4	AK158538.1 AK043711.1 BC076581.1 AK013792.1
		**1B**	Obcml-005 ENSMUST00000073822	XM_006510438.1 XM_006510437.1 XM_006510435.1 XM_006510434.1	AK040178.1 AK028345.1 AK047122.1
Homo sapiens	***OPCML***	**1A**	OBCML-003 ENST00000524381	NM_001012393.1 XM_006718846.1	BX537377.1 EU562296.1 AK299908.1
		**1B**	OBCML-001 ENST00000331898	NM_002545.3 XM_005271575.2 XM_005271574.2	EU562295.1 AK314077.1 AK289695.1 L34774.1
Rattus norvegicus	***Opcml***	**1A**	NA	NA	NA
		**1B**	Opcml-202 NSRNOT00000090643	NM_017354.1	NA

Supplementary table S2. Sequence ID of mouse individual exons for IgLON family transcripts in Ensembl.org database

<table>
<tr><th>Trans-cript</th><th>Universal I exon</th><th>I exon 1a</th><th>I exon 1a’</th><th>I exon 1b’</th><th>I exon 1b’’</th><th>II exon</th><th>III exon</th><th>IV exon</th><th>V exon</th><th>VI exon</th><th>VII exon</th></tr>
<tr><td>Opcml 1a</td><td>-</td><td>ENSMUSE00 000831318</td><td>-</td><td>-</td><td>ENSMUSE00 001063545</td><td rowspan="2">ENSMUSE00 001253872</td><td rowspan="2">ENSMUSE00 000537415</td><td rowspan="2">ENSMUSE00 000537414</td><td rowspan="2">ENSMUSE00 000537413</td><td rowspan="2">ENSMUSE00 000537411</td><td rowspan="2">ENSMUSE00 000702148</td></tr>
<tr><td>Opcml 1b</td><td>-</td><td>-</td><td>-</td><td>ENSMUSE00 000702146</td><td>Included in previous</td></tr>
<tr><td>Ntm 1a</td><td>-</td><td>ENSMUSE00 000833622</td><td>-</td><td>-</td><td>ENSMUSE00 001269187</td><td rowspan="2">ENSMUSE00 001238973</td><td rowspan="2">ENSMUSE00 001267146</td><td rowspan="2">ENSMUSE00 001230544</td><td rowspan="2">ENSMUSE00 001206009</td><td rowspan="2">ENSMUSE00 000468149</td><td rowspan="2">ENSMUSE00 000743274</td></tr>
<tr><td>Ntm 1b</td><td>-</td><td>-</td><td>-</td><td>ENSMUSE00 000702131</td><td>Included in previous</td></tr>
<tr><td>Lsamp 1a</td><td>-</td><td>ENSMUSE00 001320921</td><td>ENSMUSE00 001335414</td><td>-</td><td>ENSMUSE00 001323945</td><td rowspan="2">ENSMUSE00 000484380</td><td rowspan="2">ENSMUSE00 000486035</td><td rowspan="2">ENSMUSE00 000485091</td><td rowspan="2">ENSMUSE00 000486891</td><td rowspan="2">ENSMUSE00 001333533</td><td rowspan="2">ENSMUSE00 001327209</td></tr>
<tr><td>Lsamp 1b</td><td>-</td><td>-</td><td>-</td><td>ENSMUSE00 000484380</td><td>Included in previous</td></tr>
<tr><td>Negr1</td><td>ENSMUSE00 000830332</td><td>-</td><td>-</td><td>-</td><td>Included in previous</td><td>ENSMUSE00 000517639</td><td>ENSMUSE00 000516641</td><td>ENSMUSE00 000515122</td><td>ENSMUSE00 000268918</td><td>ENSMUSE00 000386477</td><td>ENSMUSE00 000586102</td></tr>
<tr><td>Iglon5</td><td>ENSMUSE00 000675082</td><td>-</td><td>-</td><td>-</td><td>ENSMUSE00 000372945</td><td>ENSMUSE00 000281239</td><td>ENSMUSE00 000199291</td><td>ENSMUSE00 000199288</td><td>ENSMUSE00 000199290</td><td>ENSMUSE00 000404458</td><td>ENSMUSE00 000363785</td></tr>
</table>

Supplementary table S3. Sequence ID of mouse IgLON family proteins

Transcript	Protein Ensembl.org	Protein Uniprot.org	Protein Refseq NCBI	Alternative splicing 3' end
Opcml 1a	ENSMUSP00000110898	Q6DFY2	NP_808574	NA
Opcml 1b	ENSMUSP00000073493	G5E8G3	XP_006510500.1	NA
Ntm 1a	ENSMUSP00000074578	Q8BG33	NP_758494	Possible in 3' end
Ntm 1b	ENSMUSP00000110892	Q99PJ0	NA	Possible in 3'end
Lsamp 1a	ENSMUSP00000139667	Q8BLK3	NP_780757	Possible in 3' end
Lsamp 1b	ENSMUSP00000097349	Q3TYE5	NA	Possible in 3' end
Negr1	ENSMUSP00000073664	Q80Z24/A0A4W9	NP_001034183/NP_796248	Confirmed in 3' end
Iglon5	ENSMUSP00000103608	Q8HW98	NP_001157990	NA

Supplementary figure S4. CLUSTAL O (1.2.2) multiple sequence alignment of IgLON proteins. Underline – indicates signal peptide sequence predicted by PredGPI web service (http://gpcr2.biocomp.unibo.it/predgpi/); **# -** (red) indicates six conserved cysteine residues, one pair of residues for each immunoglobulin domain (grey); * - (asterisk) indicates positions which have a single, fully conserved residue, : – (colon) indicates conservation between groups of strongly similar properties - scoring > 0.5 in the Gonnet PAM 250 matrix and **: -** (period) indicates conservation between groups of weakly similar properties - scoring =< 0.5 in the Gonnet PAM 250 matrix; N – (double underline, turquoise) indicates GPI anchor binding site. Output guide tree – true; Output distance matrix – true; Dealign input sequences – true; mBed-like clustering guide tree – true; mBed-like clustering iteration – true; Number of iterations – 5; Maximum guide tree iterations – 5; Maximum HMM iterations – 5

```
Opcml_1a      --------------------MYHPAYWI--V--FSATTALLFIPGVPVRSGDATFPKAMD
Opcml_1b      --------------MGVCGYLFLPWKCL--VV-VSLRLLFLVPTGVPVRSGDATFPKAMD
Ntm_1a        -------------MKTIQAKMHNSISWA--IF--TGLAALCLFQGVPVRSGDATFPKAMD
Ntm_1b        --------------MGVCGYLFLPWKCL--VV-VSLRLLFLVPTGVPVRSGDATFPKAMD
Lsamp_1a      -----------------MVGRVQPDRKQ--LPLVLLRLLCLLPTGLPVRSVDFN--RGTD
Lsamp_1b      MRTYWLHSVWVLGFFLSLFSLQVLAFWN--QPPAEVNLSPITIPGLPVRSVDFN--RGTD
Negr1         -------------MVLLAQGACCSNQWL--AA-VLLSLCSCLPAG---QSVDFPW-AAVD
Iglon5        ------------------MPPPAPGARLRLLAAAALAGLAVISRGLLSQSLEFS--SPAD
                                                         *   :*  :       *

                           #
Opcml_1a      NVTVRQGESATLRCTIDDRVTRVAWLNRSTILYAGNDKWSIDPRVIILVNTPTQYSIMIQ
Opcml_1b      NVTVRQGESATLRCTIDDRVTRVAWLNRSTILYAGNDKWSIDPRVIILVNTPTQYSIMIQ
Ntm_1a        NVTVRQGESATLRCTIDNRVTRVAWLNRSTILYAGNDKWCLDPRVVLLSNTQTQYSIEIQ
Ntm_1b        NVTVRQGESATLRCTIDNRVTRVAWLNRSTILYAGNDKWCLDPRVVLLSNTQTQYSIEIQ
Lsamp_1a      NITVRQGDTAILRCVVEDKNSKVAWLNRSGIIFAGHDKWSLDPRVELEKRHALEYSLRIQ
Lsamp_1b      NITVRQGDTAILRCVVEDKNSKVAWLNRSGIIFAGHDKWSLDPRVELEKRHALEYSLRIQ
Negr1         NMLVRKGDTAVLRCYLEDGASKGAWLNRSSIIFAGGDKWSVDPRVSISTLNKRDYSLQIQ
Iglon5        NYTVCEGDNATLSCFIDEHVTRVAWLNRSNILYAGNDRWTSDPRVRLLINTPEEFSILIT
              *  * :*:.* * * :::  :: ****** *::** *:*  **** :      ::*: * 

                         #                                         #
Opcml_1a      NVDVYDEGPYTCSVQTDNHPKTSRVHLIVQVPPQIMNISSDITVNEGSSVTLLCLAIGRP
Opcml_1b      NVDVYDEGPYTCSVQTDNHPKTSRVHLIVQVPPQIMNISSDITVNEGSSVTLLCLAIGRP
Ntm_1a        NVDVYDEGPYTCSVQTDNHPKTSRVHLIVQVSPKIVEISSDISINEGNNISLTCIATGRP
Ntm_1b        NVDVYDEGPYTCSVQTDNHPKTSRVHLIVQVSPKIVEISSDISINEGNNISLTCIATGRP
Lsamp_1a      KVDVYDEGSYTCSVQTQHEPKTSQVYLIVQVPPKISNISSDVTVNEGSNVTLVCMANGRP
Lsamp_1b      KVDVYDEGSYTCSVQTQHEPKTSQVYLIVQVPPKISNISSDVTVNEGSNVTLVCMANGRP
Negr1         NVDVTDDGPYTCSVQTQHTPRTMQVHLTVQVPPKIYDISNDMTINEGTNVTLTCLATGKP
Iglon5        QVGLGDEGLYTCSFQTRHQPYTTQVYLIVHVPARIVNISSPVAVNEGGNVNLLCLAVGRP
              :*  :  *:*  ****.**  .  *  *  :*:*  *:*  :*  :**.  :::***  .:.*  *:*  *:*

                                                    #
Opcml_1a      EPTVTWRHLSVK-GQGFVSEDEYLEISDIKRDQSGEYECSALNDV-AAPDVRKVKITVNY
Opcml_1b      EPTVTWRHLSVKEGQGFVSEDEYLEISDIKRDQSGEYECSALNDV-AAPDVRKVKITVNY
Ntm_1a        EPTVTWRHISPK-AVGFVSEDEYLEIQGITREQSGEYECSASNDV-AAPVVRRVKVTVNY
Ntm_1b        EPTVTWRHISPK-AVGFVSEDEYLEIQGITREQSGEYECSASNDV-AAPVVRRVKVTVNY
Lsamp_1a      EPVITWRHLTPL-GREFEGEEEYLEILGITREQSGKYECKAANEV-SSADVKQVKVTVNY
Lsamp_1b      EPVITWRHLTPL-GREFEGEEEYLEILGITREQSGKYECKAANEV-SSADVKQVKVTVNY
Negr1         EPVISWRHISPS-AKPFEN-GQYLDIYGITRDQAGEYECSAENDV-SFPDVKKVRVIVNF
Iglon5        EPTVTWRQLR----DGFTSEGEILEISDIQRGQAGEYECVTHNGVNSAPDSRRVLVTVNY
              **.::**::       *    :  *:*  *  *  *:*:***  :  *  *  :    ::*  :  **:

                                   #
Opcml_1a      PPYISKAKNTGVSVGQKGILSCEASAVPMAEFQWFKEDTRLATG-LDGVRIENKGRISTL
Opcml_1b      PPYISKAKNTGVSVGQKGILSCEASAVPMAEFQWFKEDTRLATG-LDGVRIENKGRISTL
Ntm_1a        PPYISEAKGTGVPVGQKGTLQCEASAVPSAEFQWFKDDKRLVEG-KKGVKVENRPFLSKL
Ntm_1b        PPYISEAKGTGVPVGQKGTLQCEASAVPSAEFQWFKDDKRLVEG-KKGVKVENRPFLSKL
Lsamp_1a      PPTITESKSNEATTGRQASLKCEASAVPAPDFEWYRDDTRI-NS-ANGLEIKSTEGQSSL
Lsamp_1b      PPTITESKSNEATTGRQASLKCEASAVPAPDFEWYRDDTRI-NS-ANGLEIKSTEGQSSL
Negr1         APTIQEIKSGTVTPGRSGLIRCEGAGVPPPAFEWYKGEKRLFNG-QQGIIIQNFSTRSIL
Iglon5        PPTITDVTSARTALGRAALLRCEAMAVPPADFQWYKDDRLLSSGSAEGLKVQTERTRSML
               * *  . .   .  *:  .  : **.  .**   *:*:: :   :   .    .*:  ::.    *  *
```

```
                              #
Opcml_1a      TFFNVSEKDYGNYTCVATNKLGNTNASITLYGPGAVIDGVNSASRALACLWLSGTFFAHF
Opcml_1b      TFFNVSEKDYGNYTCVATNKLGNTNASITLYGPGAVIDGVNSASRALACLWLSGTFFAHF
Ntm_1a        TFFNVSEHDYGNYTCVASNKLGHTNASIMLFGPGAVSEVNNGTSRRAGCIWLLPLLVLHL
Ntm_1b        TFFNVSEHDYGNYTCVASNKLGHTNASIMLFGPGAVSEVNNGTSRRAGCIWLLPLLVLHL
Lsamp_1a      TVTNVTEEHYGNYTCVAANKLGVTNASLVLFSKYAK-----TEPDSMQVIEFLHIDLKSI
Lsamp_1b      TVTNVTEEHYGNYTCVAANKLGVTNASLVLFRPGSV-RGINGSVSLAVPLWLLAASLFCL
Negr1         TVTNVTQEHFGNYTCVAANKLGTTNASLPLNPPSTAQYGITGSACDLFSCWSLALTLSSV
Iglon5        LFANVSARHYGNYTCRAANRLGASSASMRLLRPGSLE---NSAPRPPGPLTLLSALSWLW
               . **: ..:***** *:*:** :.**: *    :

Opcml_1a      FIKF--------
Opcml_1b      FIKF--------
Ntm_1a        LLKF--------
Ntm_1b        LLKF--------
Lsamp_1a      RHPLKVNPIQK-
Lsamp_1b      LSKC--------
Negr1         ISIFYLKNAILQ
Iglon5        WRM---------
```

Supplementary table S5. Percent Identity Matrix of full length IgLON proteins

	Opcml_1a	Opcml_1b	Ntm_1a	Ntm_1b	Lsamp_1a	Lsamp_1b	Negr1	Iglon5
Opcml_1a	X							
Opcml_1b	95.25	X						
Ntm_1a	72.92	70.26	X					
Ntm_1b	72.70	77.91	92.42	X				
Lsamp_1a	50.46	51.95	49.70	51.95	X			
Lsamp_1b	52.85	51.18	51.18	51.47	85.33	X		
Negr1	46.08	46.31	46.90	46.90	52.98	53.71	X	
Iglon5	49.70	50.15	48.18	49.24	45.90	47.27	40.67	X

Supplementary table S6. Percent Identity Matrix of N-terminal signal peptide sequences of IgLON proteins

	Opcml_1a	Opcml_1b	Ntm_1a	Ntm_1b	Lsamp_1a	Lsamp_1b	Negr1	Iglon5
Opcml_1a	X							
Opcml_1b	52.78	X						
Ntm_1a	58.33	42.50	X					
Ntm_1b	52.78	100.00	42.50	X				
Lsamp_1a	20.59	43.59	19.44	43.59	X			
Lsamp_1b	25.00	25.71	15.62	25.71	40.00	X		
Negr1	13.04	20.69	24.14	20.69	7.41	16.13	X	
Iglon5	20.00	20.00	20.00	20.00	30.00	32.14	0.00	X

Supplementary table S7. Percent Identity Matrix of I immunoglobulin domain of IgLON proteins

	Opcml_1a	Opcml_1b	Ntm_1a	Ntm_1b	Lsamp_1a	Lsamp_1b	Negr1	Iglon5
Opcml_1a	X							
Opcml_1b	100.00	X						
Ntm_1a	90.91	90.91	X					
Ntm_1b	90.91	90.91	100.00	X				
Lsamp_1a	59.77	59.77	59.77	59.77	X			
Lsamp_1b	59.77	59.77	59.77	59.77	100.00	X		
Negr1	55.68	55.68	52.27	52.27	63.74	63.74	X	
Iglon5	60.47	60.47	60.47	60.47	47.78	47.78	42.22	X

Supplementary table S8. Percent Identity Matrix of II immunoglobulin domain of IgLON proteins

	Opcml_1a	Opcml_1b	Ntm_1a	Ntm_1b	Lsamp_1a	Lsamp_1b	Negr1	Iglon5
Opcml_1a	X							
Opcml_1b	100.00	X						
Ntm_1a	71.08	71.08	X					
Ntm_1b	71.08	71.08	100.00	X				
Lsamp_1a	62.65	62.65	60.24	60.24	X			
Lsamp_1b	62.65	62.65	60.24	60.24	100.00	X		
Negr1	56.10	56.10	60.98	60.98	58.54	58.54	X	
Iglon5	65.00	65.00	58.75	58.75	53.75	53.75	46.84	X

Supplementary table S9. Percent Identity Matrix of III immunoglobulin domain of IgLON proteins

	Opcml_1a	Opcml_1b	Ntm_1a	Ntm_1b	Lsamp_1a	Lsamp_1b	Negr1	Iglon5
Opcml_1a	X							
Opcml_1b	100.00	X						
Ntm_1a	73.86	73.86	X					
Ntm_1b	73.86	73.86	100.00	X				
Lsamp_1a	48.28	48.28	48.28	48.28	X			
Lsamp_1b	48.28	48.28	48.28	48.28	100.00	X		
Negr1	44.83	44.83	45.98	45.98	55.81	55.81	X	
Iglon5	41.38	41.38	44.83	44.83	50.00	50.00	44.94	X

Supplementary table S10. Numerical values (mean $2^{-\Delta CT}$ ± SEM) for the IgLON expression data in the brain areas

		Lsamp				***Neurotrimin***				***Opcml***				***Kilon***		***Iglon5***	
		**1A**		**1B**		**1A**		**1B**		**1A**		**1B**					
No	**Tissue**	Average	SEM	Average	SEM	Average	SEM	Average	SEM	Average	SEM	Average	SEM	Average	SEM	Average	SEM
1	Eye	**1.468**	0.388	**0.643**	0.116	**0.266**	0.059	**1.260**	0.303	**0.046**	0.011	**0.189**	0.077	**0.101**	0.034	**0.389**	0.119
2	Olfactory bulb	**0.250**	0.147	**0.449**	0.217	**0.559**	0.313	**1.783**	0.922	**2.644**	0.649	**2.522**	0.077	0.675	0.170	**0.170**	0.150
3	Frontal cortex	**2.096**	0.806	**0.801**	0.295	**3.252**	0.873	**0.920**	0.333	**3.373**	0.691	**1.268**	0.398	**1.224**	0.447	**0.187**	0.087
4	Parietal cortex	**0.426**	0.139	**0.634**	0.193	**2.444**	0.062	**0.781**	0.204	**1.710**	0.200	**1.343**	0.310	**0.973**	0.097	**0.140**	0.085
5	Occipital cortex	**0.576**	0.192	**0.792**	0.117	**1.905**	0.150	**0.688**	0.352	**1.165**	0.688	**0.722**	0.476	**0.866**	0.382	**0.152**	0.082
6	Caudate putamen	**1.294**	0.755	**0.724**	0.397	**1.870**	0.500	**0.366**	0.067	**1.325**	0.377	**0.460**	0.280	**0.857**	0.485	**0.122**	0.058
7	Ventral striatum	**2.062**	0.479	**1.569**	0.219	**2.169**	0.475	**1.022**	0.371	**1.211**	0.184	**0.996**	0.113	**1.284**	0.574	**0.353**	0.053
8	Septum pellucidum	**0.911**	0.238	**0.959**	0.182	**1.577**	0.334	**0.344**	0.051	**1.033**	0.182	**0.406**	0.125	**0.617**	0.225	**0.067**	0.016
9	Temporal cortex and amygdala	**2.498**	0.349	**1.621**	0.380	**2.325**	0.347	**2.419**	0.456	**1.361**	0.293	**1.657**	0.333	**1.929**	0.400	**0.221**	0.006
10	Hippocampus	**2.433**	0.382	**0.938**	0.187	**0.724**	0.289	**0.290**	0.125	**2.274**	0.507	**0.164**	0.077	**0.303**	0.125	**0.034**	0.044
11	Thalamus	**2.121**	0.883	**1.783**	0.291	**3.347**	1.482	**0.956**	0.292	**2.030**	0.937	**0.930**	0.223	**0.897**	0.553	**0.450**	0.137
12	Hypothalamus	**2.317**	0.331	**1.697**	0.096	**1.663**	0.228	**0.908**	0.107	**1.717**	0.167	**1.428**	0.257	**0.454**	0.179	**0.178**	0.026
13	Pituitary gland	**0.232**	0.161	**0.183**	0.040	**0.025**	0.022	**0.166**	0.128	**0.019**	0.007	**0.048**	0.024	**0.710**	0.229	**0.069**	0.040
14	Midbrain and colliculi	**1.297**	0.592	**1.308**	0.622	**1.357**	0.800	**0.391**	0.237	**1.067**	0.532	**0.802**	0.352	**0.276**	0.152	**0.201**	0.063
15	Cerebellum	**0.713**	0.211	**1.571**	0.116	**1.556**	0.872	**4.611**	1.893	**0.250**	0.226	**3.448**	1.090	**1.236**	0.101	**0.216**	0.114
16	Pons	**1.229**	0.494	**1.289**	0.614	**0.986**	0.497	**0.448**	0.116	**0.551**	0.335	**1.048**	0.543	**0.292**	0.093	**0.429**	0.144
17	Medulla oblongata	**1.893**	0.959	**1.452**	0.591	**2.008**	0.582	**0.463**	0.209	**1.604**	0.608	**1.029**	0.345	**0.398**	0.062	**0.395**	0.182
18	Medulla spinalis	**0.648**	0.058	**0.864**	0.142	**1.298**	0.137	**0.156**	0.057	**0.549**	0.115	**0.434**	0.077	**0.184**	0.017	**0.275**	0.031

Supplementary table S11. Numerical values (mean $2^{-\Delta CT} \pm$ SEM) for the IgLON expression data in non-neural tissues

		Lsamp				***Neurotrimin***				***Opcml***				***Kilon***		***Iglon5***	
		**1A**		**1B**		**1A**		**1B**		**1A**		**1B**					
	**Tissue**	Average	SEM	Average	SEM	Average	SEM	Average	SEM	Average	SEM	Average	SEM	Average	SEM	Average	SEM
1	Sceletal muscle			**0.0912**	0.109					**0.1169**	0.008			**0.1985**	0.159	**0.1065**	0.030
2	Heart			**0.4292**	0.100	**0.0245**	0.013	**0.0630**	0.025			**0.0206**	0.010	**0.0422**	0.009	**0.0269**	0.006
3	Lung									**0.0200**	0.016			**0.0353**	0.023	**0.0241**	0.019
4	Liver	**0.0037**	0.001	**0.0024**	0.001			**0.0025**	0.001	**0.0020**	0.001			**0.0177**	0.013		
5	Small intestine			**0.0029**	0.002					**0.0033**	0.001	**0.0082**	0.005	**0.0037**	0.002	**0.0074**	0.005
6	Adrenal glands	**0.0532**	0.013	**0.0621**	0.013	**0.0024**	0.001	**0.0029**	0.001	**0.0056**	0.004	**0.0099**	0.003	**0.1025**	0.054	**0.0089**	0.004
7	Kidney	**0.0020**	0.001	**0.0042**	0.002	**0.0188**	0.012	**0.0025**	0.001	**0.0020**	0.001	**0.0020**	0.000	**0.0191**	0.017	**0.0320**	0.012
8	Male ureter	**0.0730**	0.068	**0.0407**	0.000					**0.0368**	0.000	**0.0217**	0.010	**0.0272**	0.023	**0.0365**	0.001
9	Ductus deferens	**0.0105**	0.008	**0.0041**	0.000			**0.0018**	0.000	**0.0062**	0.000	**0.0035**	0.000	**0.0102**	0.010	**0.0016**	0.002
10	Testis	**0.0013**	0.001	**0.0135**	0.007	**0.0047**	0.003	**0.0050**	0.001	**0.0039**	0.002	**0.0082**	0.004	**0.0227**	0.016	**0.1504**	0.052
11	Ovary	**0.0261**	0.008	**0.0196**	0.008	**0.0659**	0.013	**0.0084**	0.006	**0.0115**	0.001			**0.0260**	0.001	**0.0812**	0.020
12	Uterus	**0.0147**	0.000	**0.0415**	0.000	**0.0726**	0.055	**0.0581**	0.022	**0.0305**	0.004	**0.0155**	0.000	**0.1087**	0.010	**0.0772**	0.021

Supplementary figure S12. Illustrative histograms depicting gene expression data measured by qPCR in the brain areas and selected non-neural tissues

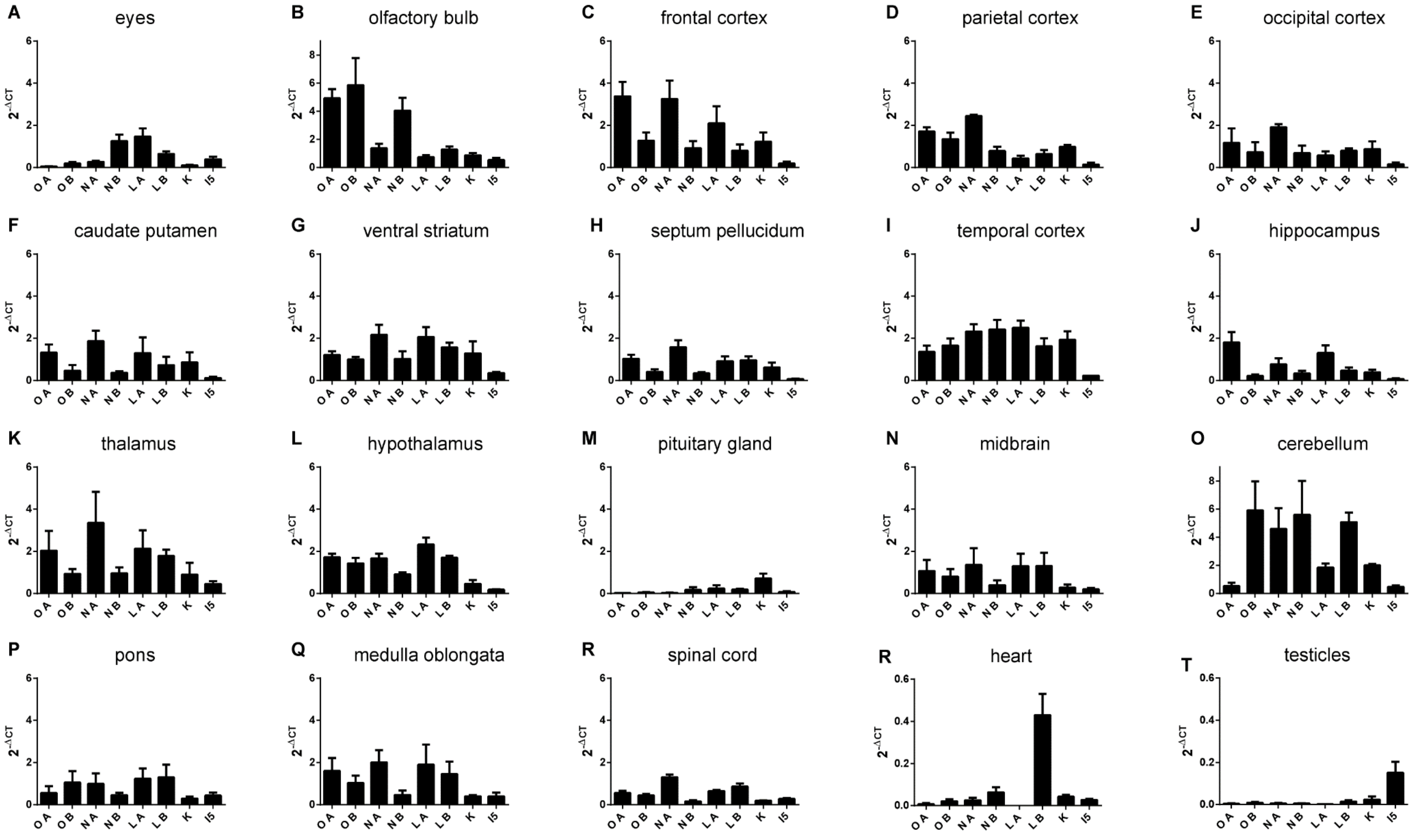

Supplementary table S13. The grouping of Wt and mutant mice expression data for statistical analysis

Grouping			
**Group I**	Wt	$Lsamp^{-/-}$	$Lsamp^{-/-}/Ntm^{-/-}$
**Group II**		$Ntm^{-/-}$	

Supplementary table S14. IgLON family expression levels in frontal cortex. Numerical values are presented as $2^{-\Delta CT}$ (mean ± SEM)

	N	***Wt***		***Lsamp$^{-/-}$***		***Ntm$^{-/-}$***		***Lsamp$^{-/-}$/Ntm$^{-/-}$***	
		Mean	SEM	Mean	SEM	Mean	SEM	Mean	SEM
Opcml 1A	6	3.50	0.27	2.37	0.26	3.52	0.44	3.39	0.40
Opcml 1B	6	1.33	0.16	1.02	0.12	1.43	0.24	1.16	0.16
Ntm 1A	6	3.45	0.31	2.01	0.19				
Ntm 1B	6	0.97	0.14	0.80	0.11				
Lsamp 1A	6	2.35	0.24			2.36	0.27		
Lsamp 1B	6	0.87	0.11			0.84	0.09		
Negr1	6	1.28	0.19	0.82	0.07	1.21	0.05	1.06	0.13
IgLON5	6	0.20	0.04	0.15	0.02	0.15	0.03	0.24	0.03
SYP	6	11.42	1.06	10.16	0.81	13.87	1.59	13.74	1.10

Supplementary table S15. IgLON family expression levels in hippocampus. Numerical values are presented as $2^{-\Delta CT}$ (mean ± SEM)

	N	***Wt***		***Lsamp$^{-/-}$***		***Ntm$^{-/-}$***		***Lsamp$^{-/-}$/Ntm$^{-/-}$***	
		Mean	SEM	Mean	SEM	Mean	SEM	Mean	SEM
Opcml 1A	6	2.19	0.14	2.38	0.29	2.32	0.26	2.67	0.35
Opcml 1B	6	0.21	0.03	0.19	0.02	0.19	0.02	0.23	0.05
Ntm 1A	6	0.81	0.09	0.97	0.11				
Ntm 1B	6	0.36	0.04	0.42	0.05				
Lsamp 1A	6	1.48	0.16			1.63	0.15		
Lsamp 1B	6	0.45	0.05			0.50	0.02		
Negr1	6	0.40	0.03	0.39	0.03	0.38	0.02	0.39	0.03
IgLON5	6	0.06	0.00	0.08	0.01	0.07	0.01	0.05	0.00
SYP	6	12.39	0.65	14.52	2.45	21.49	1.29	16.25	1.54

Supplementary figure S16. Western blot images of NTM protein expression in the frontal cortex tissue samples from Lsamp$^{-/-}$ and Wt mice. The membrane was incubated with mouse anti-Ntm (1:200) (sc-390941, Santa Cruz) or rabbit anti-GAPDH (1:10K)

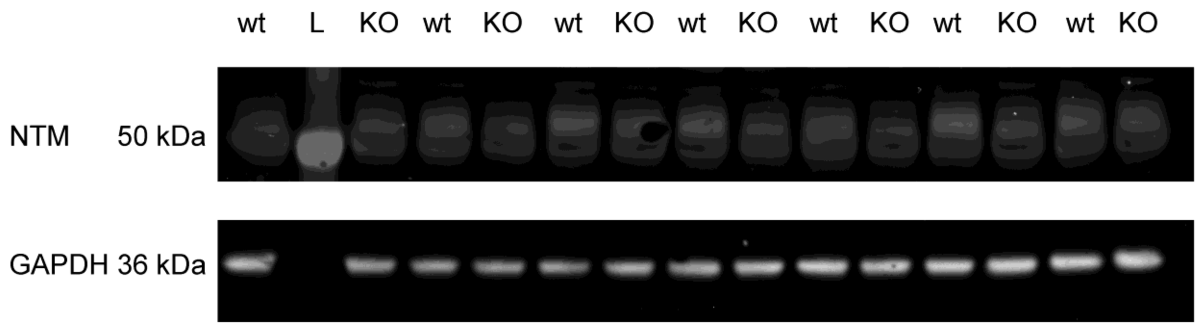

Supplementary figure S17. Western blot images of NEGR1 protein expression in the frontal cortex tissue samples from Lsamp$^{-/-}$ and Wt mice. The membrane was incubated with mouse anti-Negr1 (1:200) (sc-393293, Santa Cruz) and rabbit anti-GAPDH (1:10K)

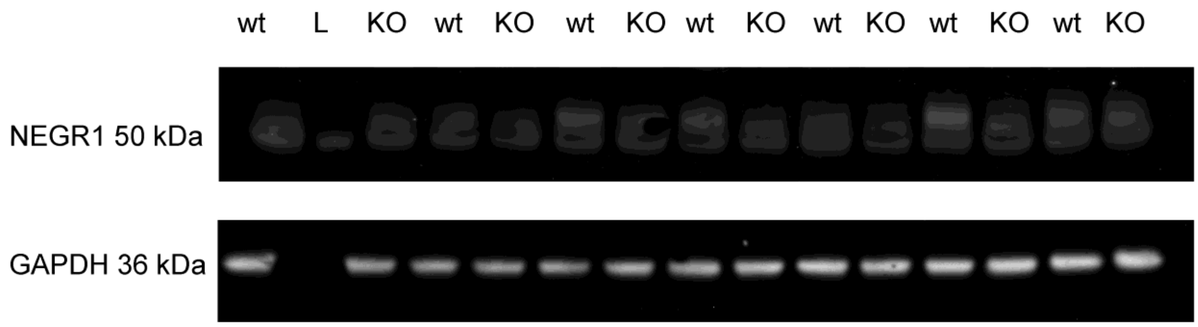

Supplementary figure S18. Pearson correlation matrix of isoform expression levels for IgLONs in the frontal cortex of wild-type mice, n=6. Statistically significant correlation coefficients $p<0.05$ have been noted in bold on right. Invidual results have been shown on scatter blot on left. OA – *Opcml* 1a, OB – *Opcml* 1b, NA – *Ntm* 1a, NB – *Ntm* 1b, LA – *Lsamp* 1a, LB – *Lsamp* 1b, N1 – *Negr1*, I5 – *Iglon5*

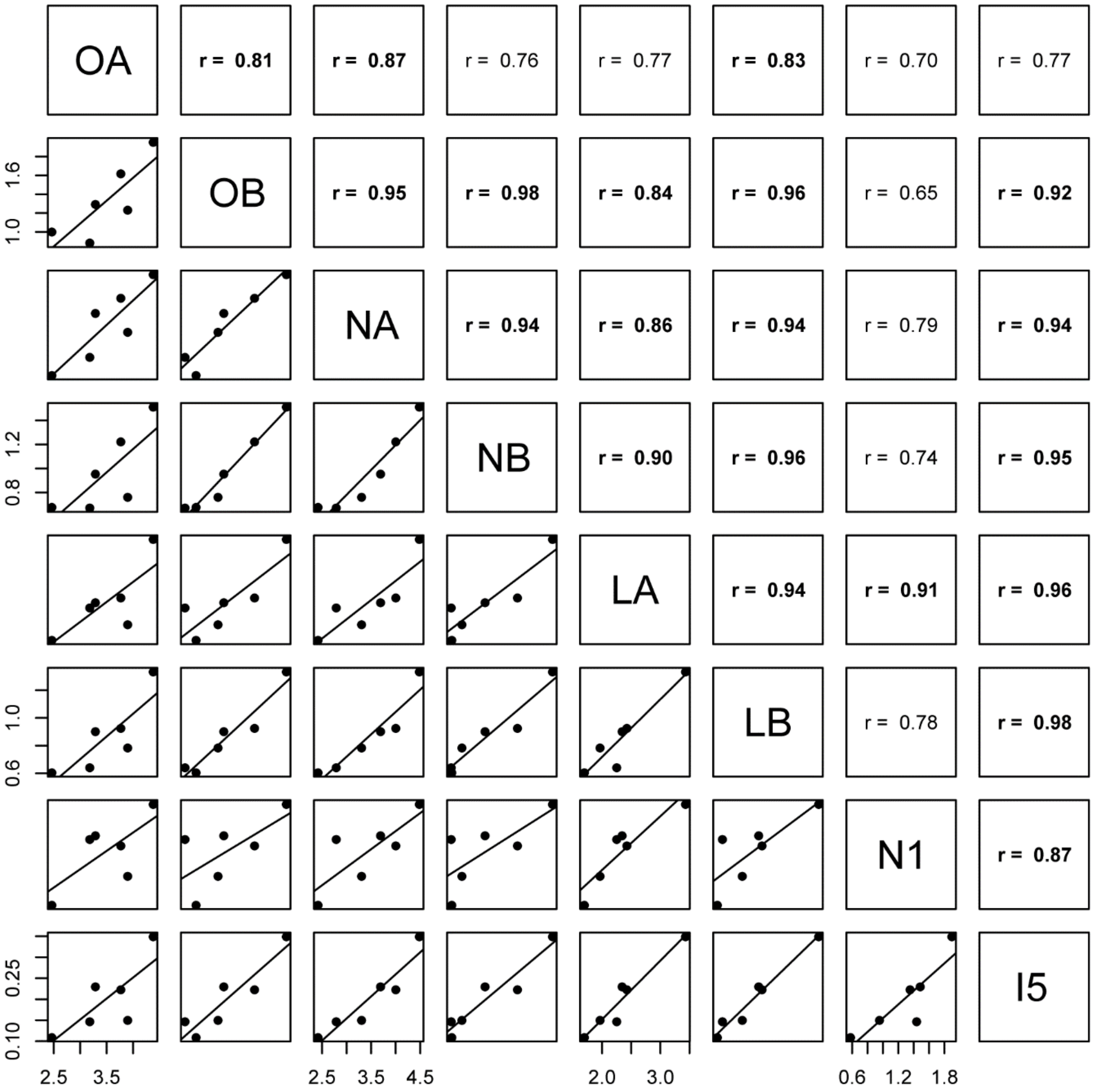

Supplementary figure S19. Pearson correlation matrix of isoform expression levels for IgLONs in the hippocampus of wild-type mice, n=6. Statistically significant correlation coefficients p<0.05 have been noted in bold. Invidual results have been shown on scatter blot on left. OA – *Opcml* 1a, OB – *Opcml* 1b, NA – *Ntm* 1a, NB – *Ntm* 1b, LA – *Lsamp* 1a, LB – *Lsamp* 1b, N1 – *Negr1*, I5 – *Iglon5*

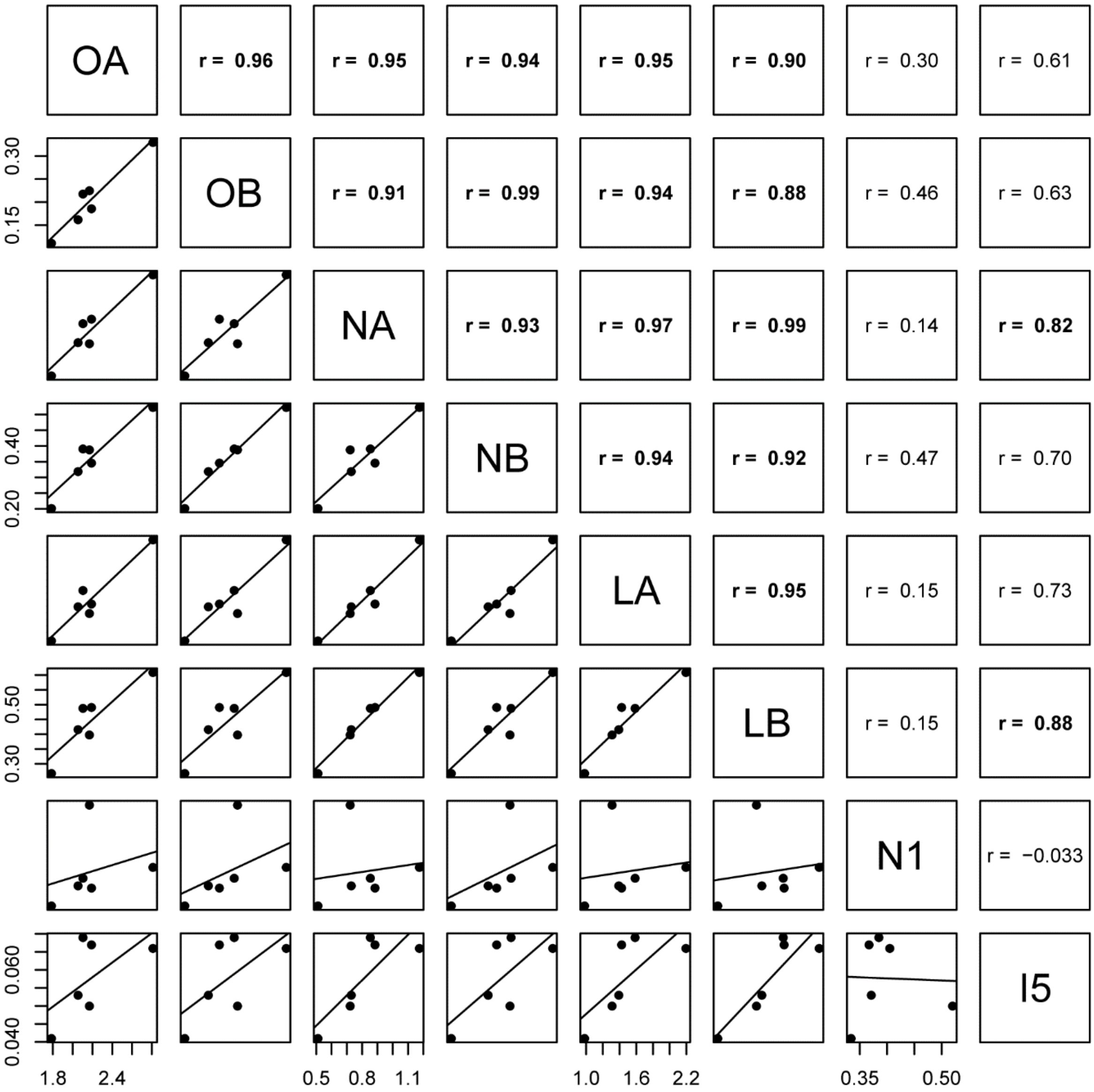

Supplement: Supplementary file 1 [file DataSheet1.pdf]
